# MLL2 Is Required in Oocytes for Bulk Histone 3 Lysine 4 Trimethylation and Transcriptional Silencing

**DOI:** 10.1371/journal.pbio.1000453

**Published:** 2010-08-17

**Authors:** Claudia V. Andreu-Vieyra, Ruihong Chen, Julio E. Agno, Stefan Glaser, Konstantinos Anastassiadis, A. Francis Stewart, Martin M. Matzuk

**Affiliations:** 1 Department of Pathology and Immunology, Baylor College of Medicine, Houston, Texas, United States of America; 2 Genomics, BioInnovationsZentrum, Technische Universitaet Dresden, Dresden, Germany; 3 Walter and Eliza Hall Institute, Melbourne, Australia; 4 Center for Regenerative Therapy, Technische Universitaet Dresden, Dresden, Germany; 5 Department of Molecular and Cellular Biology, Baylor College of Medicine, Houston, Texas, United States of America; 6 Department of Molecular and Human Genetics, Baylor College of Medicine, Houston, Texas, United States of America; The Babraham Institute, United Kingdom

## Abstract

During gametogenesis and pre-implantation development, the mammalian epigenome is reprogrammed to establish pluripotency in the epiblast. Here we show that the histone 3 lysine 4 (H3K4) methyltransferase, MLL2, controls most of the promoter-specific chromatin modification, H3K4me3, during oogenesis and early development. Using conditional knockout mutagenesis and a hypomorph model, we show that *Mll2* deficiency in oocytes results in anovulation and oocyte death, with increased transcription of p53, apoptotic factors, and *Iap* elements. MLL2 is required for (1) bulk H3K4me3 but not H3K4me1, indicating that MLL2 controls most promoters but monomethylation is regulated by a different H3K4 methyltransferase; (2) the global transcriptional silencing that preceeds resumption of meiosis but not for the concomitant nuclear reorganization into the surrounded nucleolus (SN) chromatin configuration; (3) oocyte survival; and (4) normal zygotic genome activation. These results reveal that MLL2 is autonomously required in oocytes for fertility and imply that MLL2 contributes to the epigenetic reprogramming that takes place before fertilization. We propose that once this task has been accomplished, MLL2 is not required until gastrulation and that other methyltransferases are responsible for bulk H3K4me3, thereby revealing an unexpected epigenetic control switch amongst the H3K4 methyltransferases during development.

## Abstract 2

Conditional knockout mouse strategies identify the histone methyltranferase MLL2 as a key player in epigenetic reprogramming of female gametes.

## Author Summary

It is well established that gametes and early mammalian embryos undergo extensive epigenetic changes, which are changes in phenotype or gene expression that do not entail changes in DNA sequence. However, the machinery responsible for epigenetic modification in these situations is poorly understood. In mice, we conditionally deleted the histone 3 lysine 4 (H3K4) methyltransferase *Mll2*, an enzyme that alters DNA structure and packaging, either in gametes or in somatic cells of the ovary and also produced a mouse hypomorph expressing low levels of MLL2. We show that MLL2 is required in oocytes during gametogenesis and is also needed as a maternally derived factor during early development. Oocytes deficient in *Mll2* display decreased methylation of H3K4 (H3K4me3) and show abnormal maturation and gene expression, in particular of pro-apoptotic factors. In addition, we demonstrate that embryonic genome activation is compromised in the absence of *Mll2*. Together our results identify MLL2 as one of the key players in the epigenetic reprogramming required for female fertility in the mouse.

## Introduction

Mammalian epigenomes are fundamentally reprogrammed during gametogenesis and pre-implantation development to establish the ground state of pluripotency in the epiblast cells of the blastocyst [1]–[5]. Despite the importance of this epigenetic reprogramming, how such changes are achieved is not well understood. Epigenetic reprogramming of the maternal genome occurs during oogenesis. Oocytes develop synchronously from birth until puberty, during which time they are arrested in meiotic prophase I, increase in size, and are transcriptionally active until the peri-ovulatory stage, when they undergo global transcriptional silencing [6],[7]. Transcription of the oocyte genome serves to establish the reservoirs of maternal components that are required for the first stages of embryonic development [8]. Global transcriptional silencing in oocytes is thought to be required for the efficient resumption and completion of meiosis [9] and occurs parallel to large-scale chromatin condensation and rearrangement around the nucleolus to establish a chromatin configuration termed SN (surrounded nucleolus) [1],[10],[11]. Previous studies showed that global transcriptional silencing can occur without the establishment of the SN state [1],[12]. Further studies indicated that both the acquisition of the SN configuration and transcriptional silencing are pre-requisites to achieve full embryonic developmental potential [13],[14].

Chromatin and epigenetic changes during oocyte development include histone variant exchange, alterations of DNA methylation, and global shifts in histone post-translational modifications. For instance, maternal-specific genomic imprints are established on a locus-by-locus basis [15]–[19], the linker histone 1 variant H1FOO is incorporated into chromatin [20],[21], and the global levels of 5-methyl-cytosine (CpG DNA methylation) and histone 4 acetylation at lysine 5 and 12 (H4K5 and H4K12) increase [22]. In addition, the levels of di- and tri-methylation of histone 3 at lysines 4 and 9 (H3K4me2/3 and H3K9me2/3), which are associated with active and repressed gene expression, respectively [23],[24], both increase and peak at the peri-ovulatory stage [22].

After fertilization and the second meiotic division, and between the late 1-cell and 2-cell embryo stages in the mouse, the zygotic genome is activated (ZGA) [25]–[27]. The maternal and paternal genomes of mouse 1-cell embryos display asymmetric distributions of histone H3 variants as well as di- and tri-methylation of H3K9, H3K4, H3K27, and H3K20, whereas H3K4 and H3K20 monomethylation levels are similar between both pronuclei [28]–[31]. The asymmetry in H3K9 methylation has been proposed to protect the female pronucleus from global CpG demethylation, which takes place in the paternal pronucleus soon after fertilization [29],[30]. In contrast, the significance of the asymmetry of other histone modifications remains unclear.

Histone 3 tail modifications are central to epigenetic regulation, and given the extent of epigenetic reprogramming that takes place during gametogenesis and early development, it is likely that mechanisms regulating H3 methylation play significant roles. However, few functional relationships have been identified. For instance, loss of the H3K27 methyltransferase Enhancer of Zeste 2 (EZH2) causes early post-implantation embryonic lethality [32]. The absence of maternal EZH2 results in neonatal growth retardation despite the rescue of embryonic lethality by transcription from the paternal allele [33]. This suggests a role for EZH2 in oogenesis and/or very early development. Furthermore, Embryonic Ectoderm Development (Eed), which is a subunit of the EZH2 H3K27 methyltransferase complex PRC2, is involved in genomic imprinting [34], indicating that EZH2 and H3K27 methylation play, at least, one role in epigenetic reprogramming. In most other cases studied so far, the embryonic lethality caused by loss of an H3 methyltransferase activity has precluded the analysis of how they function in gametogenesis and epigenetic reprogramming.

Tri-methylation of H3K4 marks promoters in all eukaryotes, whereas di-methylation and mono-methylation are associated with transcribed regions and active chromatin [23],[35]. In mammals, H3K4 methylation is mediated by at least six enzymes related to fly Trithorax an d the original H3K4 methyltransferase, yeast Set1 [36]. These include MLL1 (Mixed lineage leukemia 1) and MLL2 [37],[38]. Different H3K4 methyltransferases display some overlapping functions; however, they also have unique functional aspects, since MLL1 and MLL2 are both required for development [36]. MLL2 is also required for spermatogenesis, as revealed by ubiquitous conditional mutagenesis in adult males [39].

Here we report that MLL2 is required for female fertility. Using conditional knockout mutagenesis of *Mll2* in oocytes or ovarian granulosa cells, we found that MLL2 is required for several processes during oocyte postnatal development but is dispensable for granulosa cell function. Surprisingly, we found that MLL2 is essential for bulk H3K4 methylation in peri-ovulatory oocytes, indicating that MLL2 function cannot be compensated by other H3K4 methyltransferases in this cell type. We found that mice with reduced expression of *Mll2* (hypomorph allele, *Mll2^tm2^*) showed decreased fertility and a similar, albeit milder phenotype than that of oocyte-specific cKO mice. *Mll2^tm2/tm2^* females demonstrate pre-implantation embryonic lethality likely due to impaired ZGA. Our results favor the hypothesis that MLL2 is required during post-natal oogenesis and early preimplantation development, thereby identifying MLL2 as one of the key players in the epigenetic reprogramming required for female fertility in the mouse.

## Results

### Oocyte and Granulosa Cell-Specific Conditional Knockouts

**Figure 1:**
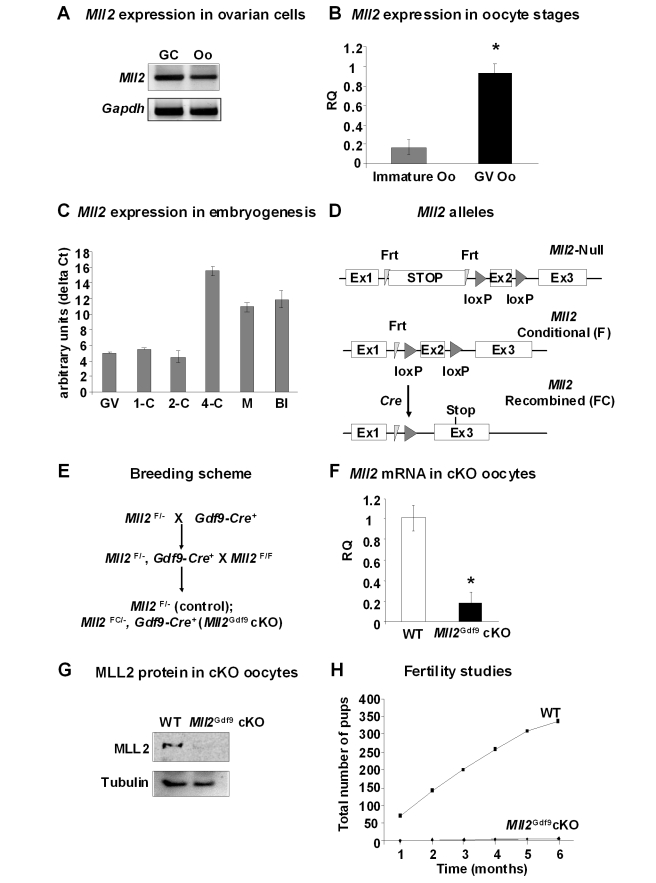
*Mll2* oocyte-specific deletion mediated by the *Gdf9*-Cre allele leads to female infertility and premature follicle loss. (A) RT-PCR analysis of *Mll2* showed expression in ovarian granulosa cells (GC) and peri-ovulatory (GV) oocytes (Oo) as determined. (B) Real time PCR (QPCR) analysis of meiotically incompetent (immature) oocytes from 12-d-old females and mature peri-ovulatory (GV, germinal vesicle) oocytes showed a significant increase in *Mll2* mRNA levels towards the peri-ovulatory stage (Student's *t* test, * *p*<0.05; three pools of oocytes were used in the analysis; *n* = 3). Results are shown relative to *Mll2* levels in GV oocytes (RQ). *Gapdh* was used as endogenous control. (C) QPCR analysis of preimplantation embryos revealed that *Mll2* is present in 1-cell embryos, likely as a maternal product, and also in subsequent embryonic stages up to the blastocyst (Bl) stage. *Gapdh* was used as endogenous control. Results are shown as arbitrary units (delta CT: CT values corrected by the endogenous control). Abbreviations: GV, germinal vesicle oocytes; 1-C, 1 cell embryos; 2-C, 2 cell embryos; 4-C, 4 cell embryos; M, morula; Bl, blatocyst. (D) cKO mice were generated by tissue-specific deletion of the *Mll2* floxed (“F”) allele in the *Mll2* null allele background. The “F” allele was generated after FLP recombination to remove the stop cassette. Cre recombination eliminates the second exon, thereby causing a frame-shift (“FC” allele). (E) Breeding scheme used to generate *Mll2^Gdf9^* cKO mice. (F) *Mll2^Gdf9^* cKO oocytes show a significant reduction in *Mll2* mRNA levels (Student's *t* test, * *p*<0.05). Results are shown as means ± S.E. relative to WT (RQ) (three oocyte pools were used; *n* = 3), and *Gapdh* was used as endogenous control. (G) *Mll2^Gdf9^* cKO oocytes show negligible levels of MLL2 protein as assessed by Western blot analysis; tubulin was used as loading control. (H) *Mll2^Gdf9^* cKO females were infertile; the cumulative number of pups over a 6-mo period is shown (*n* = 10).

The epigenome in the female pronucleus of zygotes is established in oocytes [29], under both autonomous and ovarian control [14]. We first evaluated MLL2 expression in ovarian cells and during embryogenesis by PCR analysis. The results show that both ovarian granulosa cells and oocytes from wild type (WT) females express *Mll2* (Figure 1A). QPCR analysis of meiotically incompetent (immature) and peri-ovulatory oocytes revealed a 5-fold increase in Mll2 expression as oocytes approach ovulation, suggesting a potential role for MLL2 during fertilization and/or early embryogenesis (Figure 1B; *n* = 3; Student's *t* test; *p*<0.05). *Mll2* mRNA was present in 1-cell embryos, likely as a maternal product, and it was expressed from the 2-cell stage to the blastocyst stage as determined by QPCR analysis (Figure 1C).

We used a floxed conditional allele (*Mll2^F^*) to dissect MLL2 function (Figure 1D). Previous studies with this allele and a tamoxifen-inducible Cre mouse line showed that near-ubiquitous loss of *Mll2* in adult males led to sterility [39]. Here we show that near-ubiquitous loss of *Mll2* in adult females also leads to sterility (Figure S1), further suggesting roles for *Mll2* in gametogenesis. Because *Mll2* is expressed in both granulosa cells and oocytes, we used cell-type specific conditional mutagenesis of the conditional *Mll2* allele. Conditional knockout (cKO) mice were generated using Cre recombinase driven by the growth differentiation factor 9 (*Gdf9*) and zona pellucida (*Zp3*) promoters (Figure 1E), which are expressed in oocytes [40],[41]) or by the anti-Mullerian hormone receptor 2 (*Amhr2*) promoter, which is expressed primarily in ovarian granulosa cells in adult females [42]. It is noteworthy that expression of *Gdf9-Cre* in the oocyte occurs in all follicular stages from postnatal day 3, whereas expression of *Zp3-Cre* begins at postnatal day 5 from the primary follicular stage onwards [40].

### *Mll2* Deletion Mediated by *Gdf9-Cre* Results in Female Sterility and Premature Ovarian Follicle Loss

The effect of Cre recombination on *Mll2* expression in oocytes was examined by QPCR analysis. Oocytes from *Mll2^FC/^* ^−^,*Gdf9-Cre^+^* females (herein called *Mll2^Gdf9^* cKO) displayed an 80% decrease in *Mll2* mRNA expression compared to WT controls (Figure 1F). Cre recombination deletes exon 2 causing a frame-shift and thus no functional protein is produced. Concomitantly, mRNA levels fall presumably due to nonsense mediated decay [43]. This was reflected by the presence of negligible levels of protein in isolated peri-ovulatory oocytes from *Mll2^Gdf9^* cKO females (Figure 1F). Next, by repeated breeding to males of known fertility, we found that *Mll2^Gdf9^* cKO females were sterile (Figure 1G). To begin to understand the basis of the infertility displayed by *Mll2^Gdf9^* cKO mice, we analyzed serum hormone levels. Eight-week-old *Mll2^Gdf9^* cKO females displayed increased serum gonadotropin hormones (FSH: WT, 4.24±0.43 ng/ml; *Mll2^Gdf9^* cKO, 23.81±1.99 ng/ml; and LH: WT, 0.14±0.03 ng/ml; *Mll2^Gdf9^* cKO, 0.87±0.17 ng/ml; *n* = 5; *p*<0.05) and decreased serum estradiol (WT, 21.66±3.2 ng/ml; *Mll2^Gdf9^* cKO, 12.24±1.0 ng/ml; *n* = 5; *p* = 0.06), suggesting abnormal folliculogenesis.

**Figure 2:**
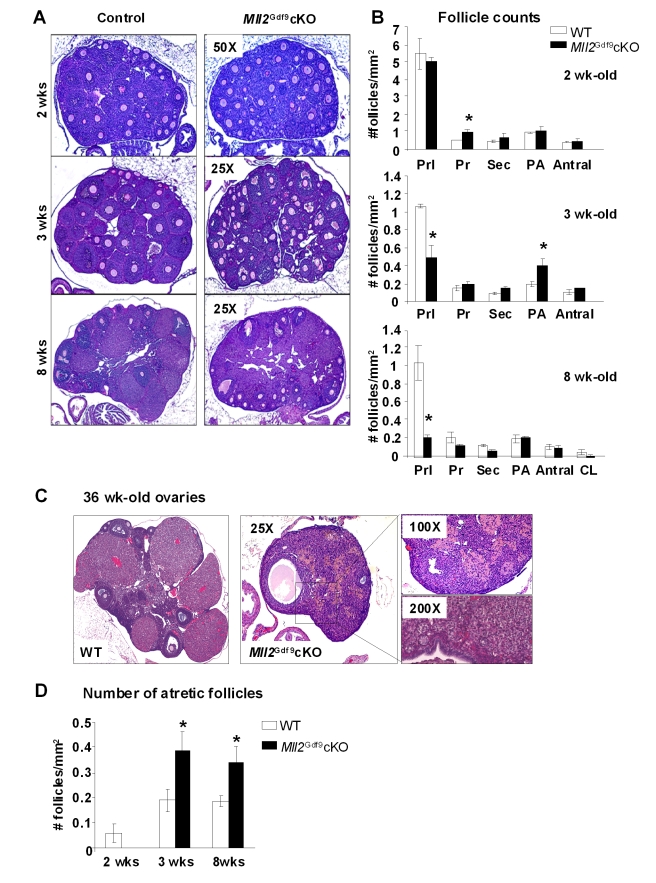
*Mll2^Gdf9^* cKO females show increased ovarian follicular recruitment and loss. (A) PAS-stained ovaries from 2-, 3-, and 8-wk-old mice; the number of follicles was greatly reduced in ovaries from 8-wk-old *Mll2^Gdf9^* cKO mice. (B) Follicle counts from 2-, 3-, and 8-wk-old mice. At 2 wk, *Mll2^Gdf9^* cKO ovaries showed significantly increased (ANOVA test, * *p*<0.05) primary follicles (Pr), whereas 3-wk-old *Mll2^Gdf9^* cKO ovaries showed significantly reduced primordial follicles (Prl) and increased preantral follicles (PA) (ANOVA test, * *p*<0.05). *Mll2^Gdf9^* cKO ovaries from 8-wk-old mice showed further reduction in Prl follicles (ANOVA test, * *p*<0.05). Means of follicle counts corrected by surface area ± S.E. are shown (ovarian sections from five individual females were used; *n* = 5). Abbreviations: Prl, primordial; Pr, primary; Sec, secondary; A, antral; CL, corpora lutea. (C) *Mll2^Gdf9^* cKO ovaries from 36-wk-old mice stained with hematoxylin-eosin showed very few follicles (boxed area). (D) From 3 wk onwards, the number of atretic follicles was significantly higher in *Mll2^Gdf9^* cKO ovaries, compared to controls. Means of follicle counts corrected by surface area ± S.E. are shown (ovarian sections from five individual females were used; *n* = 5). ANOVA test, * *p*<0.05.

To investigate further this potential defect, histological sections of ovaries from *Mll2^Gdf9^* cKO females of various ages were evaluated and follicle counts were performed. Based on morphology [44], follicles were grouped into three categories: dormant (primordial), growing (primary, secondary, preantral, and antral), or dying (atretic and zona pellucida remnants). Two-week-old *Mll2^Gdf9^* cKO ovaries displayed a higher number of primary growing follicles (Figure 2A and 2B, upper panels), suggesting increased follicular recruitment. Three-week-old *Mll2^Gdf9^* cKO ovaries showed a 50% decrease in dormant follicles and an increase in growing preantral follicles (Figure 2A and 2B, middle panels), whereas 8-wk-old *Mll2^Gdf9^* cKO ovaries displayed an 80% reduction in dormant follicles (Figure 2A and 2B, lower panels), demonstrating increased follicle loss. Further, the number of atretic follicles was significantly increased in *Mll2^Gdf9^* cKO females from 3 wk of age onwards (Figure 2D; *n* = 5, ANOVA test, *p*<0.05). Older (36-wk-old) *Mll2^Gdf9^* cKO ovaries showed few follicles (Figure 2C). Together, the results suggest that increased early follicle recruitment and oocyte loss contributes to infertility of *Mll2^Gdf9^* females.

### *Mll2^Gdf9^* cKO Females Fail to Ovulate

**Figure 3:**
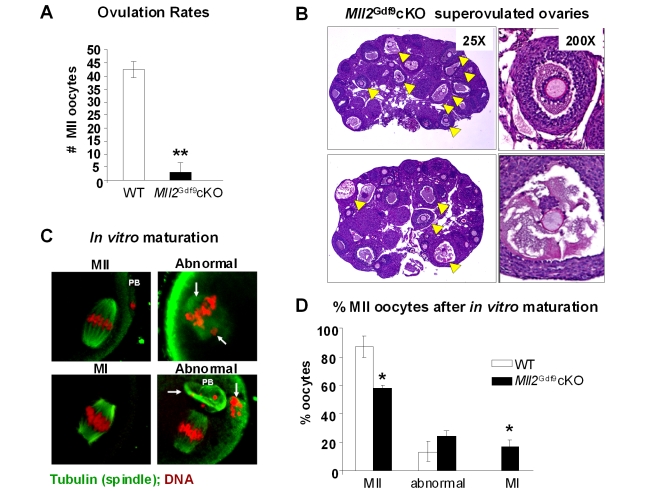
*Mll2^Gdf9^* cKO females show impaired ovulation. (A) Ovulation rates were significantly reduced in 3-wk-old superovulated *Mll2^Gdf9^* cKO females (Student's *t* test, ** *p*<0.01). Means ± S.E. are shown (five individual females were used; *n* = 5). (B) PAS-stained ovaries from superovulated 3-wk-old *Mll2^Gdf9^* cKO mice showed trapped oocytes (yellow arrowheads), which correlated with decreased ovulation rates in this mouse line. (C) In vitro oocyte maturation studies; representative single plane confocal laser microscopy micrographs of peri-ovulatory oocytes cultured for 16 h to allow resumption of meiosis; arrows show lagging chromosomes. Magnification: 800×. Abbreviations: MI, meiosis I; MII, meiosis II; PB, polar body. (D) Percentages of meiosis I (MI), meiosis II (MII), or abnormal oocytes after 16 h in culture (ANOVA test, * *p*<0.05). Means ± S.E. of four independent experiments are shown; a total of 120 oocytes per genotype were analyzed.

By stimulation of pre-pubertal mice with gonadotropin hormones to induce ovulation and resumption of meiosis, we found that *Mll2^Gdf9^* cKO females were essentially anovulatory (Figure 3A; *n* = 4, Student's *t* test, *p*<0.05). Anovulation was confirmed by histologycal analysis of ovaries from hormonally stimulated females, which showed oocytes trapped in luteinizing structures (Figure 3B). To determine whether resumption of meiosis was also compromised, we cultured isolated peri-ovulatory oocytes and found that the majority of *Mll2^Gdf9^* cKO oocytes progressed to meiosis II, whereas a small but significant fraction remained arrested in meiosis I (Figure 1C and 1D; ANOVA test; *p*<0.05).

### *Mll2^Gdf9^* cKO Oocytes Fail to Establish Transcriptional Repression and Show Abnormal Histone Tail Modifications

**Figure 4:**
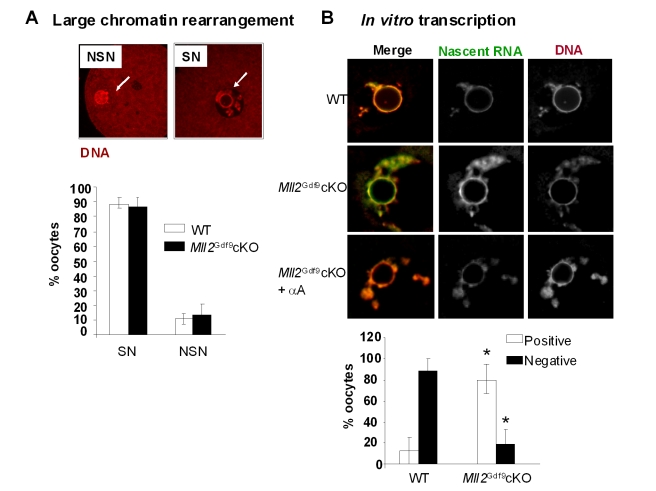
*Mll2^Gdf9^* cKO oocytes fail to establish transcriptional repression. (A) Confocal laser microscopy analysis of peri-ovulatory oocytes stained with propidium iodide to visualize DNA (upper panel); oocytes were scored as displaying a surrounded nucleolus (SN) or a non-surrounded nucleolus (NSN) configuration (lower panel). Arrows show the nucleolus. No significant differences were observed between controls and *Mll2^Gdf9^* cKO oocytes (ANOVA test, * *p* = 0.08). Means ± S.E. are shown; a total of 170 oocytes from five individual females were analyzed in three independent experiments. Magnification: 800×. (B) Run-on and confocal microscopy analyses of Br-UTP labeled nascent RNA in peri-ovulatory oocytes. Upper panel: Representative single plane confocal micrographs are shown as merge and split channels (Br-UTP, green staining; and DNA: propidium iodide, red staining). Lower panel: oocytes were scored as positive or negative for Br-UTP staining; note that *Mll2^Gdf9^* cKO oocytes were transcriptionally active (ANOVA test, * *p*<0.05). Transcription was RNA Pol II dependent as it was abrogated by α-amanitin (αA) (magnification: 800×). Means ± S.E. are shown; a total of 90 oocytes from four individual females were evaluated in three independent experiments.

During folliculogenesis, oocytes acquire a number of epigenetic marks [22],[45], undergo large-scale chromatin rearrangement from a non-surrounding (NSN) to a surrounded (SN) nucleolus configuration, and establish a state of global transcriptional repression [14]. Confocal microscopy analysis showed that the percentage of peri-ovulatory oocytes from WT and *Mll2^Gdf9^* cKO females that acquired the expected SN configuration [13] was comparable (Figure 4A). However, using a run-on assay based on Br-UTP incorporation into nascent RNA, we found that, unlike controls, 80% of the *Mll2^Gdf9^* cKO oocytes were still transcriptionally active at the peri-ovulatory stage (Figure 4B, upper and lower panels; Student's *t* test, *p*<0.05). Furthermore, treatment with α-amanitin reduced Br-UTP incorporation [46], indicating that transcription was RNA Pol II-dependent (Figure 4B). Hence, our results show that MLL2 is required for oocyte transcriptional silencing and confirm that the establishment of the SN configuration does not depend on the acquisition of global transcriptional repression [1].

**Figure 5:**
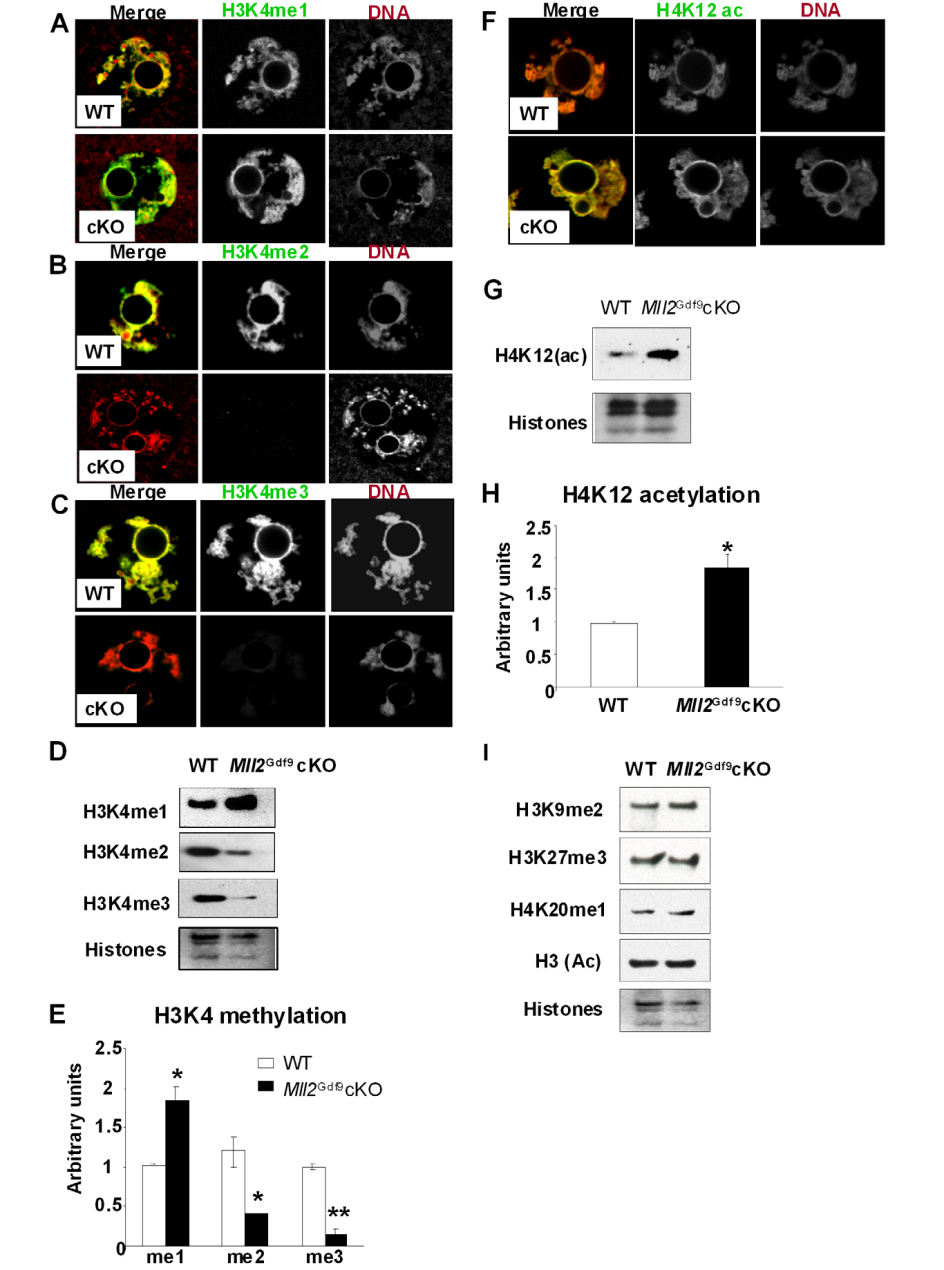
*Mll2^Gdf9^* cKO oocytes show loss of bulk H3K4me2 and H3K4me3 as well as H4 hyperacetylation. (A–C) Confocal microscopy micrographs showing H3K4 methylation levels in peri-ovulatory oocytes. (A) An increase in H3K4me1 is apparent in *Mll2^Gdf9^* cKO oocytes (lower panel) compared to control (upper panel). In contrast, a decrease in H3K4me2 (B) and H3K4me3 (C) levels was observed in *Mll2^Gdf9^* cKO oocytes (B and C, lower panels) compared to controls (B and C, upper panels). Magnification: 800×. Representative single plane micrographs are shown as merge and split channels of histone tail modifications (green) and DNA (red). (D–E) Western blot analysis of H3K4 methylation in chromatin fractions from peri-ovulatory oocytes. (D) Representative micrographs of Western blots showing H3K4me1, H3K4me2, and H3K4me3 levels. (E) Chemoluminescence quantification revealed a significant increase in global H3K4me1 and a significant decrease in global H3K4me2 and H3K4me3 levels in *Mll2^Gdf9^* cKO oocytes; Student's *t* test, * *p*<0.05; ** *p*<0.01. Means ± S.E are shown. Three pools of 100 oocytes each from 3–4 females per genotype were used in three independent experiments; samples were normalized against total histones. (F) Confocal microscopy micrographs showing H4K12 acetylation levels in peri-ovulatory oocytes. An increase in H4K12 acetylation is apparent in *Mll2^Gdf9^* cKO oocytes (lower panel) compared to controls (upper panel). Magnification: 800×. Representative single plane micrographs are shown as merge and split channels of histone tail modifications (green) and DNA (red). (G–H) Western blot analysis of H4K12 acetylation (H3K12(ac)) in chromatin fractions from peri-ovulatory oocytes. (G) Representative micrographs of Western blots showing H4K12 acetylation levels. (H) Chemoluminescence quantification revealed a significant increase in global H4K12 acetylation in *Mll2^Gdf9^* cKO oocytes; Student's *t* test, * *p*<0.05; Means ± S.E are shown. Three pools of 100 oocytes each from 3–4 females per genotype were used in three independent experiments; samples were normalized against total histones. (I) Western blot analysis of other histone tail modifications in chromatin fractions from peri-ovulatory oocytes. Representative micrographs of Western blots showing H3K9me2, H3K27me3, H4K20me1, and H3(ac); total histones were used as internal loading controls. No changes were observed in the levels of any of the histone tail modifications analyzed. Three pools of 100 oocytes each from 3–4 females per genotype were used in three independent experiments.

Histone tail lysines can be mono-(me1), di-(me2), or tri-(me3) methylated [35]. In particular, H3K4me3 is found at the promoters of genes that are transcriptionally active or poised for activity [47]–[50]. Because MLL2 is a H3K4 histone methyltransferase, we next analyzed the methylation status of H3K4. Confocal microscopy analysis of *Mll2^Gdf9^* cKO oocytes showed an apparent increase in inmunostaining of H3K4me1 (Figure 5A) and a decrease in immunostaining of H3K4me2 and H3K4me3 (Figure 5B and 5C), whereas control oocytes displayed the expected distribution of methylation marks (Figure 5A–C) [51]. Western blot analysis of chromatin fractions followed by chemoluminescence quantification confirmed that *Mll2^Gdf9^* cKO oocytes have significantly lower levels of H3K4me2 and H3K4me3 and significantly higher levels of H3K4me1 (Figure 5D and 5E; Student's *t* test; *p*<0.05). Interestingly, confocal microscopy and Western blot analyses showed that acetylated H4K12, a modification associated with active chromatin [52], was also significantly increased in *Mll2^Gdf9^* cKO oocytes (Figure 5F–5H; Student's *t* test; *p*<0.05). In contrast, *Mll2^Gdf9^* cKO oocytes displayed global levels of repressive marks such as H3K9me3, H3K27me3, and H4K20me1, or those of acetylated H3 that were comparable to those of controls (Figure 5I).

Together, the results suggest that MLL2 is responsible for the majority of maternal H3K4me3 and that other histone methyltransferases cannot compensate MLL2 function in this cell type. Loss of *Mll2* does not affect the establishment/maintenance of other histone post-translational modifications. Importantly, our results show that *Mll2* deficiency leads to a decrease in H3K3me3 but not H3K4me1, suggesting that a histone methyltransferase other than MLL2 is responsible for this methylation mark. The results also indicate that transcriptional repression in peri-ovulatory oocytes is defective in the absence of MLL2. It is unclear whether this phenomenon is a consequence of altered H3K4 methylation, defects in granulosa cell-oocyte communication, or other factors. However, the increased H4K12 acetylation likely reflects the lack of transcriptional silencing observed in *Mll2^Gdf9^* cKO oocytes.

### Abnormal Expression Pro-Apoptotic Genes and *Iap* Elements in *Mll2^Gdf9^* cKO Oocytes

Elevated transcription in *Mll2^Gdf9^* cKO peri-ovulatory oocytes prompted further investigation of potential gene expression changes. A survey of selected genes involved in oocyte function [53]–[59] and early embryogenesis [60]–[63] by QPCR analysis of peri-ovulatory oocytes revealed no changes in the majority of genes investigated (Table 1). An exception was the B-type cyclin E3 ubiquitin ligase cyclin B1 interacting protein 1 (*Ccnb1ip*, also known as *Hei10*), which is important for both meiosis and embryogenesis [64]. *Ccnb1ip/Hei10* expression was significantly decreased in *Mll2^Gdf9^* cKO oocytes (*p*<0.05; Table 1). Interestingly, expression of the histone 4 acetylase *Kat5/Tip60* (Lysine acetyltransferase 5), which has been previously reported to be present in oocytes, was significantly increased in *Mll2^Gdf9^* cKO oocytes (Table 1, *p*<0.05) [65] and may therefore contribute to the observed increase in H4K12 acetylation.

**Table 1:** Real time PCR analysis of molecular changes in *Mll2^Gdf9^*cKO oocytes.

Gene	Control	cKO
**Epigenetic factors**		
*Mll3*	1.02±0.26	0.70±0.22
*Setd1a*	1.03±0.28	1.11±0.17
*Setd8*	1.03±0.16	1.07±0.15
*Smyd3*	1.06±0.05	0.87±0.18
*Wdr5*	1.01±0.10	1.07±0.30
*Ash2l*	1.01±0.11	1.08±0.30
*Rbbp5*	**0.92±0.04**	**2.27±0.34** a
*Uhrf1*	**0.94±0.07**	**0.43±0.04** a
*Tip60/Kat5*	**1.01±0.07**	**8.53±2.03** a
**Oocyte function and meiosis**		
*Gdf9*	1.00±0.12	1.06±0.18
*Bmp15*	1.69±0.36	1.43±0.72
*Pten*	1.00±0.10	1.53±0.35
*Kit*	0.87±0.11	0.76±0.06
*Yx2*	0.99±0.10	0.94±0.07
*Pde3a*	0.99±0.11	1.29±0.51
*Ccnb1ip1*	**1.11±0.12**	**0.04±0.01** a
Apoptosis/p53		
*Bcl2*	1.02±0.17	0.91±0.09
*Casp6*	**1.00±0.08**	**1.46±0.04** a
*Bax*	**1.06±0.17**	**1.92±0.18** a
*Cdkn1a/p21*	**1.06±0.06**	**2.07±0.37** a
*Fos*	**1.37±0.43**	**6.29±0.57** a
*Bbc3/Puma*	1.23±0.23	1.91±0.34
*Setd7*	**1.05±0.07**	**1.87±0.27** a
*p53*	**1.15±0.10**	**2.12±0.20** a
**Development**		
*Nalp5*	1.10±0.47	1.03±0.50
*Dpp3a*	1.07±0.22	0.98±0.23
*Smarca4*	1.07±0.18	0.85±0.31

Real-time PCR analysis of peri-ovulatory cKO oocytes showed differential expression of genes involved in various biological processes. Results are shown as the mean ± S.E. from three independent oocyte pools (100 oocytes per pool, from 3–4 individual females per genotype). Data were analyzed using the non-parametric Mann-Whitney U test.

a *p*<0.05.

QPCR analysis also showed that *Mll2^Gdf9^* cKO oocytes overexpressed several apoptosis-associated genes (Table 1), including p53 (transformation related protein 53; TRP53) and *Setd7* (SET domain lysine methyltransferase 7, also know as *Set7/9*) (Table 1 and Figure S2A), which methylates p53 and prevents its degradation [66]. P53 has been implicated in prenatal oocyte apoptosis, and peri-ovulatory oocytes express low p53 levels [67]. Consequently, it is possible that up-regulated SETD7 stabilizes p53 in *Mll2^Gdf9^*cKO oocytes leading to increased oocyte death. Supporting this hypothesis, we found high levels of p53 protein (Figure S2B) and upregulation of several pro-apoptotic p53 targets, including *Bax* (Bcl2-associated X protein), *Cdkn1a* (cyclin-dependent kinase inhibitor 1A; P21), *Fos* (FBJ murine osteosarcoma viral oncogene homolog) [68],[69], and *Casp6* (caspase 6) [70],[71] in *Mll2^Gdf9^* cKO oocytes (Figure S2C and Table 1). Since follicular atresia is thought to occur via apoptosis, the increase in expression of pro-apoptotic factors is in line with the increase in the number of atretic follicles observed in *Mll2^Gdf9^* cKO ovaries.

Finally, because of the widespread lack of transcriptional repression in *Mll2^Gdf9^* cKO oocytes, we looked at the expression of retrotransposon interspersed repeats by QPCR analysis. We found that intracisternal A particle (*Iap*) elements were abnormally transcribed in *Mll2^Gdf9^* cKO peri-ovulatory oocytes, whereas LINE-1 and SINE-1 elements were not (Figure S3A). Increased *Iap* expression in *Mll2^Gdf9^* cKO oocytes correlated with hypomethylation of *Iap* DNA loci, as evaluated by methylation specific primers (Figure S3B) and bisulphite sequencing (Figure S3C). However, *Mll2^Gdf9^* cKO oocytes showed a strong signal for 5-methyl-cytosine staining, indicating that loss of CpG methylation was not a widespread phenomenon (Figure S3D). Because retrotransposons drive the expression of multiple host genes in female gametes [72], *Iap* up-regulation could contribute to loss of *Mll2^Gdf9^* cKO oocytes. Absence of *Uhrf1* (ubiquitin-like, containing PHD and RING fingers 1 also known Np95) in cell lines and mouse embryos has been associated with increased transcription of interspersed repeats due to DNA hypomethylation [73]. Consequently, we examined *Uhrf1* expression levels and found a 2-fold decrease (Table 1), suggesting a role in *Iap* deregulation. Decreased *Uhrf1* in *Mll2^Gdf9^* cKO oocytes could also contribute to *Cdkn1a* deregulation [74]. Together, the results suggest that stabilization of p53 and abnormal expression of its downstream targets as well as deregulated expression of retrotransposable elements may trigger apoptosis in *Mll2^Gdf9^* cKO oocytes, leading to oocyte death and, ultimately, premature follicle loss and sterility.

### Deletion of *Mll2* Using *Zp3-Cre*

**Figure 6:**
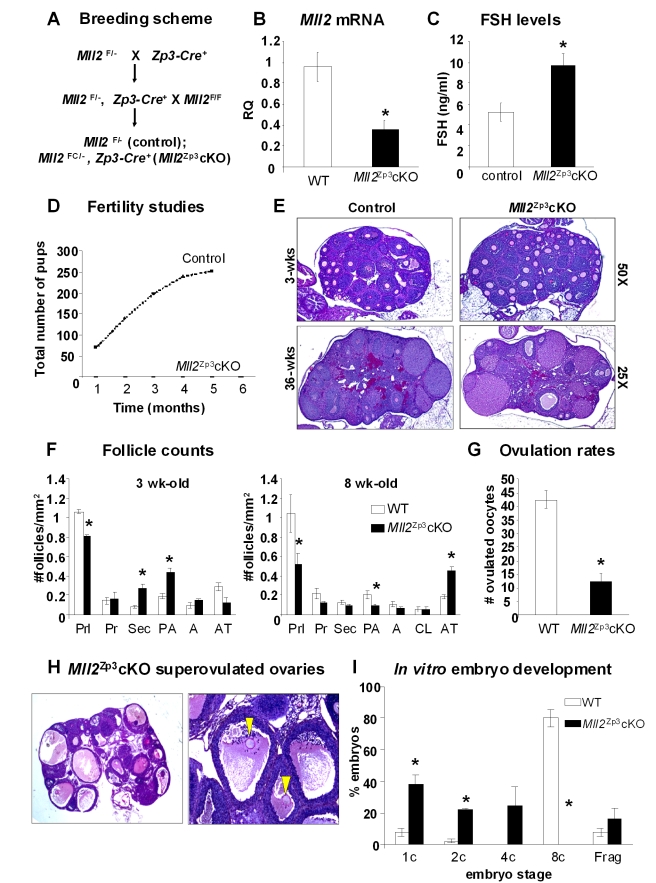
Oocyte-specific deletion of *Mll2* mediated by the *Zp3-Cre* allele. (A) Breeding scheme used to generate *Mll2^Zp3^*cKO mice. (B) *Zp3*-cKO oocytes show a significant reduction in *Mll2* mRNA levels (Student's *t* test, * *p*<0.05). Results are shown as means ± S.E. relative to WT (RQ) (three oocyte pools were used; *n* = 3) and *Gapdh* was used as endogenous control. (C) FSH levels were significantly higher in serum samples from 8-wk-old *Mll2^Zp3^* cKO females compared to controls (Student's *t* test, * *p*<0.05; *n* = 10). (D) Fertility studies shown as cumulative number of pups over a 6-mo period. *Mll2^Zp3^*cKO females were infertile. (G) Ovulation rates were significantly reduced in *Mll2^Zp3^*cKO females compared to controls (WT) (Student's *t* test; * *p*<0.05; *n* = 5). (E) PAS-stained ovaries from 3- and 36-wk-old mice. Original magnification: 25× and 50×; note that different from *Mll2^Gdf9^* cKO mice, *Mll2^Zp3^* cKO ovaries still contain follicles at 36 wk of age. (F) Follicle counts in *Mll2^Zp3^* cKO ovaries. *Mll2^Zp3^* cKO ovaries had a significantly lower number of primordial follicles and increased numbers of secondary and multilayer preantral follicles 3 wk of age (left panel). By 8 wk of age (right panel), the number of primordial and multilayer preantral follicles is significantly reduced and the number of atretic follicles is increased in *Mll2^Zp3^* cKO ovaries. (ANOVA test; * *p*<0.05; ovarian sections from five females were used in the analysis; *n* = 5). Abbreviations: Prl, primordial; Pr, primary; Sec, secondary; PA, preantral; A, antral; CL, corpora lutea; Atr, atretic. (I) Cultures of embryos from *Mll2^Zp3^* cKO females crossed to WT males showed a developmental arrest between the 1-cell and the 4-cell stages. Results are presented as the % embryos (average) ± standard error from five independent experiments. ANOVA test; * *p*<0.05. A total of 95 embryos from six females per genotype were used in three independent experiments. Abbreviations: C, cell; Frag, fragmented; WT, control wild type.

**Figure 7:**
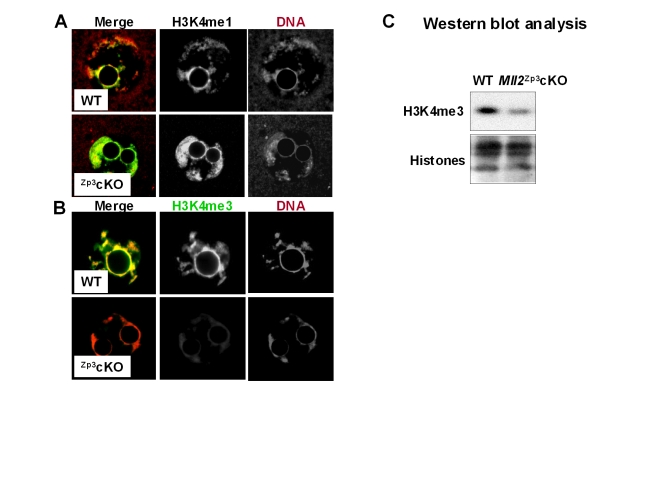
*Mll2^Zp3^* cKO oocytes show decreased H3K4 tri-methylation. (A–B) Confocal microscopy micrographs showing H3K4 methylation levels in peri-ovulatory oocytes. (A) An increase in H3K4me1 is apparent in *Mll2^Zp3^* cKO oocytes (lower panel) compared to control (upper panel). In contrast, a decrease in H3K4me3 (B) levels was observed in *Mll2^Zp3^* cKO oocytes (lower panel) compared to controls (upper panel). Magnification: 800×. Representative single plane micrographs are shown as merge and split channels of histone tail modifications (green) and DNA (red). (C) Representative micrographs of Western blots showing H3K4me3 levels in chromatin fractions from *Mll2^Zp3^* cKO peri-ovulatory oocytes. A decrease in global H3K4me3 is apparent in *Mll2^Zp3^*c KO oocytes (Student's *t* test, * *p*<0.05). Three pools of 100 oocytes each from 3–4 females per genotype were used in three independent experiments. Total histones were used as internal loading controls.

To further substantiate the phenotype observed in the *Mll2^Gdf9^* cKO mouse model, we employed conditional mutagenesis using the *Zp3-Cre* allele (Figure 6A). Peri-ovulatory oocytes from *Mll2^FC/^* ^−^, *Zp3Cre^+^* females (herein called *Mll2^Zp3^* cKO) show a significant decrease in *Mll2* expression as evaluated by QPCR analysis (Figure 6B; Student's *t* test; *p*<0.05; *n* = 3). Similar to *Mll2^Gdf9^* cKO mice, *Mll2^Zp3^* cKO females showed a significant increase in FSH levels (Figure 6C) and were infertile (Figure 6D). However, *Mll2^Zp3^* cKO females showed a milder rate of follicule loss than that of *Mll2^Gdf9^* cKO mice, as evidenced by the lack of significant changes in the number of atretic follicles at 3 wk of age (Figure 6E and 6F) and the presenc of follicles at 36 wk of age (Figure 6E). In contrast to *Mll2^Gdf9^* cKO females, which were anovulatory, *Mll2^Zp3^*cKO mice displayed an ovulation rate of 20%–25% compared to controls (Figure 6G). Consistent with decreased ovulation rates, *Mll2^Zp3^* cKO ovaries showed a number of oocytes trapped in luteinizing structures (Figure 6H). Interestingly, oocytes ovulated by *Mll2^Zp3^* cKO females were capable of fertilization *in vivo*, and the majority of embryos generated from matings of *Mll2^Zp3^* cKO females and WT males became developmentally arrested between the 1-cell and 2-cell stages (Figure 6I). Confocal microscopy analysis of *Mll2^Zp3^* cKO oocytes showed an increase in the levels of H3K4me1 (Figure 7A) and a reduction in the levels of H3K4me3 (Figure 7B) comparable to that observed in *Mll2^Gdf9^* cKO oocytes (Figure 5A–C). Western blot analysis confirmed the decrease in bulk H3K4me3 in *Mll2^Zp3^*cKO oocytes (Figure 7C). These results demonstrate that *Mll2^Zp3^* cKO females display a similar but slightly milder phenotype than *Mll2^Gdf9^* cKO females, consistent with the later onset of Cre expression in the *Zp3-Cre* line [40]. Importantly, the results show that MLL2 is required not only during oogenesis but also for early embryogenesis.

### *Mll2* Deletion in Granulosa Cells Has Limited Impact on Female Fertility

**Figure 8:**
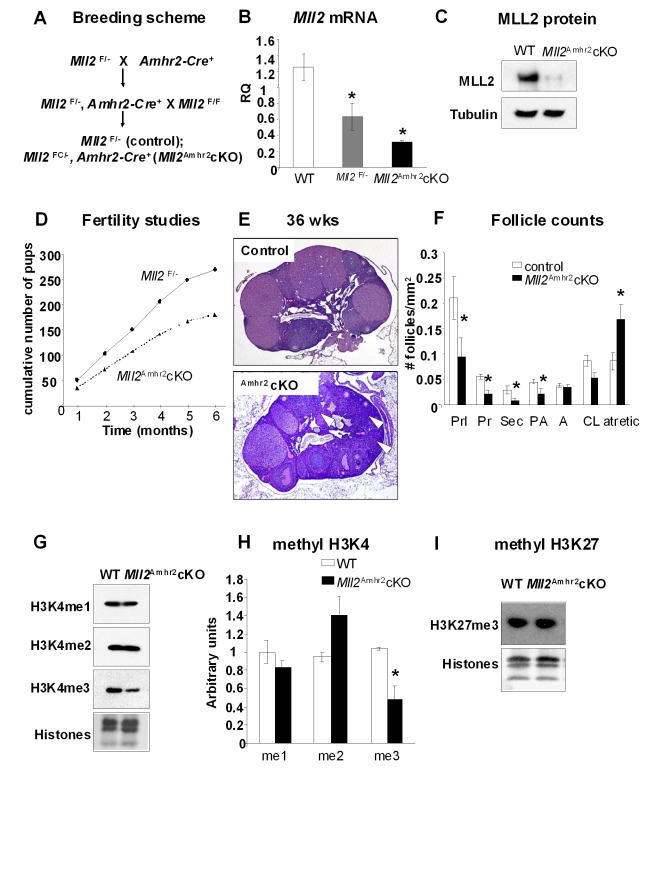
MLL2 is not required for granulosa cell function. (A) Breeding scheme used to generate granulosa cell-specific cKO mice (*Mll2^Amhr2^* cKO). (B) QPCR analysis of *Mll2* levels in granulosa cells from wild type (WT), *Mll2^F/^* ^−^ (heterozygous control), and *Mll2^Amhr2^* cKO females (granulosa cells from three individual females were used in the analysis; *n* = 3). *Mll2^Amhr2^* cKO granulosa cells show a significant decrease in the levels of *Mll2*, whereas *Mll2^F/^* ^−^ granulosa cells show the expected 50% decrease in *Mll2* levels (ANOVA test; * *p*<0.05). *Gadpdh* was used as internal control. (C) Western blot analysis of *Mll2^Amhr2^* cKO granulosa cells showed decreased MLL2 protein levels. Tubulin was used as loading control. (D) Fertility studies shown as cumulative number of pups over a 6-mo period. *Mll2^Amhr2^* cKO females displayed a small reduction in the number of pups (*n* = 10). (E) PAS-stained ovaries from 36-wk-old *Mll2^Amhr2^* cKO mice showed PAS-positive zona pellucida remnants (oocyte remnants, arrowheads). Note that the number of follicles present at this age is higher than that observed in the oocyte-specific cKO mouse lines. (F) Follicle counts in 36-wk-old ovaries. *Mll2^Amhr2^* cKO ovaries show a significant decrease in the number of quiescent amd growing follicles and a significantly higher number of atretic follicles (ovarian sections from five females were used in this study; *n* = 5). Abbreviations: Prl, primordial; Pr, primary; Sec, secondary; PA, preantral; A, antral; CL, corpora lutea. ANOVA test; * p<0.05. (G–H) Western blot analysis of H3K4 methylation in chromatin fractions from granulosa cells. (G) Representative micrographs of Western blots showing H3K4me1, H3K4me2, and H3K4me3 levels. (H) Chemoluminescence quantification revealed a significant decrease in global H3K4me3 levels in *Mll2^Amhr2^* cKO granulosa cells (Student's *t* test, * *p*<0.05). Means ± S.E are shown. Granulosa cells from three individual females per genotype were used in three independent experiments; samples were normalized against total histones. (G) Representative Western blot analysis of the repressive mark H3K27me3 showed no global changes in *Mll2^Amhr2^* cKO granulosa cells. Total histones were used as internal loading control.

To evaluate whether the loss of MLL2 in ovarian granulosa cells affects female fertility, we generated a mouse line carrying a granulosa cell deletion of *Mll2* (*Mll2^FC/^* ^−^, *Amhr2-Cre*, herein called *Mll2^Amhr2^* cKO) (Figure 8A). In contrast to the oocyte cKOs (*Mll2^Gdf9^* cKO and *Mll2^Zp3^* cKO), the loss of *Mll2* in granulosa cells had only a small impact on fertility. *Amhr2-Cre* action on *Mll2* ^F/F^ resulted in ∼75% reduction in granulosa cell *Mll2* mRNA levels (Figure 8B) and an almost complete loss of protein in *Mll2^Amhr2^* cKO granulosa cells as expected from the conditional allele design (Figure 8C). In young mice, *Mll2* deficiency in granulosa cells had little impact on fertility (Figure 8D), ovulation or fertilization rates (*Mll2^Amhr2^*: 45.0±3.1; control: 37.0±2.5, *n* = 4), gonadotropin hormone levels (FSH: control, 6.33±1.39 ng/ml; *Mll2^Amhr2^* cKO, 9.51±2.57 ng/ml; LH: control, 0.26±0.07 ng/ml; *Mll2^Amhr2^* cKO, 0.17±0.08 ng/ml; *n* = 7), or ovarian histology (unpublished data). However, older animals showed a small reduction in fertility (Figure 8D), abnormal ovarian histology, and increased follicle loss (Figure 8E and 8F), as evidenced by a significant increase in the number of atretic follicles and a significant decrease in the number of growing follicles (Figure 8F; ANOVA test, *p*<0.05). Follicle loss correlated with increased FSH levels in *Mll2^Amhr2^* cKO mice (control, 5.31±0.82 ng/ml; *Mll2^Amhr2^* cKO, 10.83±0.83 ng/ml; *n* = 7). Analysis of H3K4 methylation levels by Western blot of *Mll2^Amhr2^* cKO granulosa cell chromatin fractions showed no changes in the levels of H3K4me1 or H3K4me2 (Figure 8G and 8H). A 50% decrease in global H3K4me3 was observed in *Mll2^Amhr2^* cKO granulosa cells (Figure 8G and 8H; Student's *t* test, *p*<0.05); however, no differences were found in the levels of H3K27me3 between WT and *Mll2^Amhr2^* cKO granulosa cells (Figure 8I). Finally, QPCR analysis of other H3K4 methyltransferases showed a significant increase in the expression of *Mll3* (WT: 1.46±0.07; *Mll2^Amhr2^* cKO: 2.48±0.47; *n* = 3, *p*<0.05) but not *Mll1* (WT: 1.08±0.08; *Mll2^Amhr2^* cKO: 1.53±0.47; *n* = 3) in *Mll2^Amhr2^* cKO granulosa cells. The results indicate that MLL2 is not essential for granulosa cell function and that an increase in *Mll3* in *Mll2^Amhr2^* cKO granulosa cells may, at least partially, compensate for *Mll2* loss.

### *Mll2* ^tm2/tm2^ Female Mice Show Reduced Fertility

We generated an *YFP-Mll2* fusion protein by gene targeting in ES cells and name the allele targeted mutation 2 (*Mll2* ^tm2^; Figure S4A). Homozygous mice carrying the *Mll2* ^tm2^ allele (*Mll2* ^tm2/tm2^) were viable (Figure S4B and S4C); however, they showed no detectable yellow fluorescent signal in any tissue (unpublished data). To determine whether *YFP-Mll2* was expressed, we analyzed *Mll2* ^tm2/tm2^ ovaries by real-time PCR and Western blot and found a significant decrease in both mRNA (Figure S4D) and protein (Figure S4E) levels, indicating that the mice carrying this allele were hypomorphs for MLL2. We next examined the fertility of *Mll2* ^tm2/tm2^ females by mating them to males of known fertility. From the first month of mating, *Mll2* ^tm2/tm2^ females showed a 50% reduction in the number of pups per litter compared to WT or *Mll2* ^tm2/+^ controls, and showed impaired cumulative fertility with age (Figure S4F). Notably, the fertility of *Mll2* ^tm2/+^ females was also cumulatively reduced (Figure S4F).

### Embryos from *Mll2* ^tm2/tm2^ Females Show Decreased In Vitro Developmental Potential

Ovaries from *Mll2* ^tm2/tm2^ females appeared normal until 4 mo of age (Figure S4G). Unlike the oocyte-specfic cKO mouse lines, ovulation rates of hormonally stimulated pre-pubertal *Mll2* ^tm2/tm2^ and control mice were comparable (*Mll2* ^tm2/+^: 42.0±3.0; *Mll2* ^tm2/tm2^:34.0±5; *n* = 4). Furthermore, fertilization rates of young control and *Mll2* ^tm2/tm2^ females were also comparable (WT: 30.0±2.7; *Mll2* ^tm2/+^: 28.0±2.7; *Mll2* ^tm2/tm2^: 29.4±3.9; *n* = 12). These findings suggested that subfertility in *Mll2* ^tm2/tm2^ females was due to embryonic defects. In embryo cultures, most embryos from *Mll2* ^tm2/tm2^ intercrosses (75%) arrested between the 1-cell and 4-cell stages whereas >70% of WT embryos developed to the blastocyst stage regardless of the male genotype (Figure S4H). This phenotype was very similar to that of embryos obtained from crossing *Mll2^Zp3^*cKO females to WT males. Additionally, a higher percentage of fragmentation (22%), which indicates decreased survival, was observed in *Mll2* ^tm2/tm2^ embryos compared to controls (5%). The severity of this outcome was slightly relieved when *Mll2* ^tm2/tm2^ females were crossed to WT males (Figure S4I), indicating that expression of *Mll2* from the paternal allele could partially rescue the defect. Development was also impaired when *Mll2* ^tm2/+^ females were mated to *Mll2* ^tm2/tm2^ or even WT males (Figure S4H and S4I).

### *Mll2* ^tm2/tm2^ Embryos Show Both Reduced ZGA and Bulk H3K4 Methylation

Because the majority of the *Mll2* ^tm2/tm2^ embryos arrested before the 8-cell stage, we evaluated their ability to undergo ZGA using synthesis of the Transcription-Requiring Complex [75] as a marker. Embryos from *Mll2* ^tm2/tm2^ intercrosses showed a 30% reduction in TRC levels compared to controls (Figure S5A and S5B; *n* = 3, *p*<0.05). Therefore, defective ZGA likely contributes to the developmental block observed in *Mll2* ^tm2/tm2^ embryos.

To investigate the potential link between MLL2 activity and ZGA, we analyzed the status of H3K4 methylation in *Mll2* ^tm2/tm2^ embryos. Immunofluorescence and confocal microscopy (Figure S6) showed decreased levels of H3K4me2 and H3K4me3 in female pronuclei of 1-cell embryos from *Mll2* ^tm2/tm2^ intercrosses (Figure S6B and S6C), whereas control embryos showed the expected asymmetry for H3K4 methylation between the female and male pronuclei (Figure S6B abd S6C) [31]. Western blot analysis of chromatin fractions and chemoluminescence quantification confirmed a significant reduction in H3K4me3 levels in *Mll2* ^tm2/tm2^ zygotes (Figure S6, left and right panels). The results from the hypomorphic allele are in agreement with and extend the results from conditional mutagenesis of *Mll2*. Near-ubiquituous mutagenesis of *Mll2* in female adults caused infertility (Figure S1). Removal of *Mll2* from oocytes but not from granulosa cells also caused infertility. *Gdf9*-cre-mediated deletion of *Mll2* caused a more severe phenotype than *Zp3*-cre-mediated deletion, consistent with the earlier expression of *Gdf9* in oocytes. The hypomorphic allele of *Mll2* presented the mildest phenotype, consistent with a reduction, but not abolishment of MLL2 protein levels (Figure S4E).

## Discussion

In this article, we identify MLL2 as one of the major factors controlling bulk H3K4 methylation during oocyte growth and pre-implantation development. Our results indicate that MLL2 is autonomously required in the oocyte for normal oogenesis and pre-implantation development. Oocyte-specific cKO females showed severe defects in fertility, which were also observed in both conditionally mutated and hypomorphic adult females, wheras granulosa cell-specific cKO females showed almost normal fertility. The results suggest differential regulation in H3K4 methylation in gametes and somatic cells. Unlike cKO oocytes (Table 1), *Mll2^Amhr2^* cKO granulosa cells showed an increase in *Mll3*, which could compensate, at least partially, for the loss of *Mll2*.

**Figure 9:**
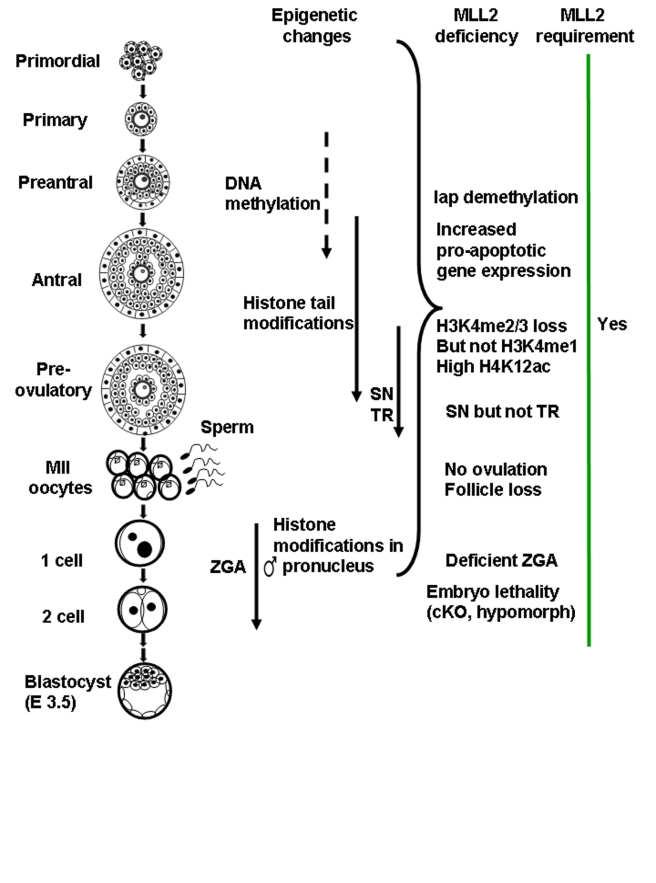
Summary of MLL2 function during gametogenesis and early embryogenesis. During normal folliculogenesis, oocytes are transcriptionally active and grow in size but do not proliferate. In contrast, the surrounding somatic cells are actively dividing. A number of epigenetic marks are acquired by oocytes during folliculogenesis, including DNA methylation and histone tail modifications. Towards the peri-ovulatory stage, oocytes undergo a large chromatin re-arrangenment into the surrounded nucleolus (SN) configuration and global transcriptional repression (TR). Upon ovulation and fertilization, the maternal pronucleus retains the majority of the histone tail modification marks established in the oocyte, whereas the male pronucleus acquires them progressively. Between the late 1-cell and the 2-cell embryo stages, the maternal mRNA and protein stores are depleted and the zygotic genome is activated (ZGA). MLL2 is expressed in oocytes and during pre-implantation development. *Mll2* oocyte-specific cKO females are infertile due to anovulation and follicle loss. *Mll2-*deficient oocytes show reduced H3K4me3 but not H3K4me1, indicating another enzyme monomethylates H3K4 in oocytes. Oocytes lacking *Mll2* also show abnormal expression of pro-apoptotic genes and Iap elements, which may contribute to oocyte death and, ultimately, follicle loss. Although *Mll2*-deficient oocytes display the SN configuration, they fail to repress transcription, likely due to loss of communication with the granulosa cell compartment. Finally, studies in the hypomorph and the *Mll2^Zp3^* cKO model reveal that MLL2 is required either directly or indirectly for embryogenesis and ZGA. Together the results suggest that MLL2 is continuously required during oogenesis and early embryonic development.

Our studies demonstrate that MLL2 is required during the transcriptionally active period of oocyte growth for the establishment and/or maintenance of bulk H3K4 tri-methylation (Figure 9). A previous study showed that H3K4 methylation increases from immature (meiotically incompetent) oocytes to peri-ovulatory stage (meiotically competent) oocytes [22]. That study also showed that the expression of the H3K4 methyltransferases *Mll1* and *Smyd3* decreased towards the peri-ovulatory stage, suggesting that a different methyltransferase is responsible for H3K4 methylation in peri-ovulatory oocytes. Here, we show that *Mll2* expression increases towards the peri-ovulatory stage and that its loss in oocytes leads to decreased H3K4 methylation. Our results are consistent with the idea that MLL2 is responsible for the increase in H3K4 methylation in peri-ovulatory oocytes observed by Kageyama and collaborators. In addition, the results suggest that although MLL2 is required throughout oogenesis, MLL2 might be particularly important as oocytes develop meiotic competence.

Changes in bulk H3K4me3 methylation (Figures 5, 7, and 9) observed in oocytes were unexpected because loss of *Mll2* in ES cells or adult mice did not affect bulk H3K4me3 [39],[76]. Our findings demonstrate that MLL2 is responsible for the majority of H3K4me3 in oocytes and that a different enzyme(s) is responsible for H3K4me1. Mono-methylation by dedicated monomethylases that co-operate with dedicated di-/tri-methylases has been observed for other histone tail lysines but not for H3K4 [35]. Until now, there has been no evidence that a similar mechanism may operate on mammalian H3K4 methylation [77], thereby differing from the paradign H3K4 methyltransferase, yeast Set1C, which mediates mono-, di-, and tri-methylation under the control of its various subunits [36],[78],[79].

Oocytes lacking *Mll2* displayed a variety of defects including lack of global transcriptional silencing at the peri-ovulatory stage and abnormal expression of *Uhfr1*, *Ccnblip1*, *Iap*, and pro-apoptotic factors (Table 1 and Figures S2, S3, and 9). The failure to undergo transcriptional silencing is paradoxical because MLL2, like other trithorax-Group factors, acts to maintain expression of target genes [39]. A possible explanation for this paradox is that MLL2 is required for expression of key factors that mediate global transcriptional silencing, and therefore, this defect is likely to be an indirect consequence of *Mll2* loss. Previous studies have shown that granulosa cell-oocyte communication is essential for transcriptional repression in oocytes [12]. Thus, it is possible that altered communication between the gamete and somatic cell compartments in *Mll2^Gdf9^* cKO ovaries, as evidenced by the ovulatory defect, may contribute to the lack of transcriptional repression. A previous study showed that global transcriptional silencing occurred without the large chromatin rearrangement into the SN configuration [1]. Here we extend this observation by showing the converse: acquisition of the SN configuration in *Mll2*-deficient oocytes occurred without global transcriptional silencing, confirming that these two events are independent.

It is likely that the observed overexpression of *Iap* elements and apoptotic factors in *Mll2-*deficient oocytes is an indirect consequence of *Mll2* loss. In contrast, the decrease in the expression of *Uhfr1* and *Ccnblip1* may reflect direct regulation of these genes by MLL2, since both genes are expressed from CpG islands, as are all MLL2 targets described to date [39].

Results in the hypomorph and *Mll2^Zp3^* cKO models demonstrated a developmental arrest between the 1- and 4-cell stages, when ZGA takes place in mouse embryos. In addition, *Mll2* hypomorph zygotes displayed impaired ZGA (Figure 9). A possible explanation for this phenotype is that unknown maternal factors required for ZGA are lacking in *Mll2*-deficient oocytes. Another possibility is that the establishment of H3K4 di- and tri-methylation in the oocyte by MLL2 contributes to establish a poised starting point for the activation of the embryonic genome after fertilization. Finally, it is also possible that MLL2 itself might be required to activate transcription in the zygote. In this context, we note that MLL2 appears to be continuously required after fertilization during at least the first three cell divisions (Figure 9). This conclusion is supported by the fact that MLL2 is expressed in pre-implantation embryos from the 2-cell stage onwards and that embryos derived from *Mll2^Zp3^* cKO and *Mll2* ^tm2/tm2^ females undergo developmental arrest. Recently, an unidentified MLL protein has been implicated as part of a BRG-1 containing complex required for ZGA [60]. Our results from the hypomorph model suggest that MLL2 could be this protein.

Finally, recent studies have shown that H3K4 histone methyltransferases, including MLL2, may also play a role in nuclear receptor activation and hormone signaling, at least in human cells [80]. Thus, defective signaling pathways could also contribute to the phenotype observed in the various *Mll2* cKO mouse lines. Undoubtely, more studies are needed to dissect the potential role of MLL2 in those pathways.

Together with previous studies [37], our findings suggest that during development there is a switch from an MLL2-dependent to MLL2-independent state. In the oocyte, MLL2 is the major tri-H3K4 methyltransferase, and maternal MLL2 is required for the first embryonic divisions. Previous studies showed that *Mll2* KO mice develop up to E9.5 [37], suggesting that an MLL2-independent state is established after ZGA. We hypothesize that a cell-type specific requirement for MLL2 may be established later in development during gastrulation. This implies a cell-type specific switch in epigenetic roles, which points to a new dimension of complexity in epigenetic regulation.

Our results indicate that MLL2 is required during post-natal oogenesis and the 2–3 embryonic cell divisions, thereby identifying MLL2 as one of the key players in the epigenetic reprogramming required for female fertility in the mouse. Given the recent interest in epigenetics raised by the reprogramming of somatic cells to a pluripotent state equivalent to the epiblast, termed iPS (induced pluripotent stem cells), it will be interesting to determine whether MLL2 is also required for the generation of iPS. If this holds true, MLL2 dependency could be used as a criterion to determine whether reprogramming to iPS cells recapitulates endogenous mechanisms or is largely synthetic short-circuiting.

## Materials and Methods

### Generation of Mouse Lines

Conditional KO mice were generated by crossing Rosa26CreERT2 [81] *Gdf9-Cre* [40], zona pellucida 3 (*Zp3*)-*Cre* [41], or anti-Mullerian hormone receptor 2 (*Amhr2*)*-Cre* [42] mice and *Mll2* ^+/−^ and *Mll2^F/F^* mice [37] as explained in Text S1. The hypomorphic line *Mll2^tm2^* was generated inadvertently when a yellow fluorescence protein (YFP) tag was added in frame with the first ATG of *Mll2* located in exon 1 (Figure 1A, Text S1). All mouse lines were maintained in the 129/C57BL/6 hybrid background. Genotyping from tail DNA was performed as described [37],[40],[42]. The decrease in *Mll2* levels was confirmed by quantitative PCR (QPCR) analysis using primers described in Table S1. Mice were maintained in accordance with the NIH Guide for the Care and Use of Laboratory Animals and under German license nr. 24-9168.11-1-2004-26.

### Fertility Studies, Serum Hormone Levels, Histological Analysis, Cell Collection, and Culture

Fertility studies, serum hormone measurements, and histological analysis were performed as previously described [82]. More details are provided in Text S1. 21-d-old females were injected with 5 IU of pregnant mare serum gonadotropin (PMSG; Calbiochem, EMD, Gibbstown, NJ), and granulosa cells or peri-ovulatory oocytes were collected 47 h later [82]. Superovulation and ovulation rates in 21-d-old females were carried out as described [82]. In vitro oocyte maturation and embryo cultures are detailed in Text S1.

### RT-PCR and Quantitative Real Time PCR (QPCR) Analyses

RNA from oocytes or granulosa cells was isolated and subjected to QPCR analysis as described in Text S1. Primer sets are described in [69] and Table S1. Primer amplification efficiency and transcript levels were calculated as previously described [82]. *Gapdh* was used as endogenous control. The relative amount of target gene expression for each sample was presented as the mean ± SEM.

### Chromatin Isolation and Western Blot Analysis

Chromatin was isolated from oocytes or granulosa cells without nuclease treatment [83] and equal amounts of protein or equal numbers of oocytes were fractionated in 4%–12% SDS-PAGE Bis-Tris gels (Invitrogen, Carlsbad, CA). Total histones were determined by SYPRO Ruby protein blot stain (Molecular Probes, Invitrogen). Proteins were detected with Super-signal West Pico-detection system (Pierce/Thermo Fisher Scientific, Pittsburgh, PA), and levels were quantified by using the Image J software (NIH). Results are presented as a ratio of modified histone to total histones or protein to tubulin and represent the average of three pools collected in three experiments.

### Immunofluorescence and Confocal Laser-Scanning Microscopy

Peri-ovulatory oocytes were fixed and stained as previously described [84]. Samples were mounted in well-slides (Fisher Scientific) using Vectashield containing propidium iodine (Vector Laboratories, Burlingame, CA). Fluorescence was detected on a laser-scanner confocal microscope (Carl Zeiss, Thornwood, NY). Images were collected at 40× magnification with zoom level equal to 2. For each experimental series, oocytes from all groups were processed under the same conditions and images were captured using the same microscope settings.

### Metabolic Labeling, TRC Complex Detection, Run-On Assays, and DNA Methylation Assays

Metabolic labeling of 2-cell embryos, protein extraction, and TRC detection were performed as previously reported [85],[86]. Transcriptional activity was determined in run-on studies after 5-bromo uridine 5′-triphosphate (BrUTP; Sigma) incorporation as previously described [12],[46]. A detailed explanation is presented in Text S1. Global DNA methylation was assessed by immunostaining against 5-methylcytosine, and *Iap* DNA methylation was determined by bisulfite genomic sequencing [73] or bisulphite conversion followed by PCR analysis using methylation-specific primers, which were designed using MethPrimer.

### Data Analysis

Statistical significance was determined by Student's *t* test or one-way analysis of variance (ANOVA) and test for multiple comparison analysis Newman-Keul's (SNK's); *p* values <0.05 were considered to be significant.

## Supporting Information

Figure S1 **Loss of** ***Mll2*** **in adult females using tamoxifen-induced Cre recombination leads to infertility.** *Mll2* ^F/F^; Rosa26CreERT2/^+^ (Mll2^F^, conditional KO, grey bars) or *Mll2* ^F/+^; Rosa26CreERT2/^+^ (control, black bars) females (upper panel) were mated to WT males. Initially ten 2-mo-old adults of each genotype were mated to establish fertility (before induction), then they were treated with tamoxifen as described [38] and tested for fertility. The WT males (one male for each pair of tamoxifen treated females) were exchanged every week. Data show the number of successful litters. Note that tamoxifen treatment provokes infertility in females regardless of genotype. After treatment was discontinued, control animals fully recover fertility, whereas *Mll2* conditional KO females remain infertile.
(0.34 MB TIF)

Figure S2 ***Mll2^Gdf9^*** **cKO oocytes show increased expression of the methyltransferase** ***Setd7*** **, p53 stabilization, and expression of p53 downstream pro-apoptotic target genes.** (A) QPCR analysis showed a significant increase in the methyltransferase *Setd7* in *Mll2^Gdf9^* cKO peri-ovulatory oocytes (Student's *t* test, * *p*<0.05; three pools of oocytes were used in the analysis (*n* = 3)). *Gapdh* was used as endogenous control. (B) Representative Western blot analysis showing increased p53 protein levels in *Mll2^Gdf9^* cKO peri-ovulatory oocytes; tubulin was used as loading control. Note that p53 is normally absent in peri-ovulatory stage oocytes. (C) QPCR analysis showed a significant increase in the expression of p53 target genes,which are involved in apoptosis, including *Bax*, *Cdkn1a*, *Fos*, and *Casp6* in *Mll2^Gdf9^* cKO peri-ovulatory oocytes (Student's *t* test, * *p*<0.05; ** *p*<0.01; three pools of oocytes were used in the analysis (*n* = 3)). *Gapdh* was used as endogenous control.
(0.18 MB TIF)

Figure S3 ***Mll2^Gdf9^*** **cKO oocytes display abnormal levels of the retrotransposable element Iap.** (A) QPCR analysis of retrotransposon mRNA in isolated peri-ovulatory *Mll2^Gdf9^*cKO oocytes showed an increase (Student's *t* test, * *p*<0.05) in *Iap* (intracisternal A particle) but not in LINE-1 (Long Interspersed Nuclear Element 1, L1) or SINE-1 (Short Interspersed Nuclear Element); means ± S.E. are shown (three pools of 100 oocytes each were used in the analysis; *n* = 3). (B) Methylation-specific PCR analysis of the *Iap* promoter showed a significant decrease in CpG DNA methylation in peri-ovulatory *Mll2^Gdf9^* cKO oocytes (Student's *t* test, * *p*<0.05); means ± S.E. are shown (three pools of 100 oocytes each were used in the analysis; *n* = 3). (C) Hypomethylation of *Iap* in peri-ovulatory *Mll2^Gdf9^* cKO oocytes was confirmed by bisulphite sequencing. Methylated and unmethylated CpGs are shown as filled or open circles, respectively. (D) 5-methylcytosine (5-Me-C) staining and confocal microscopy analysis reveal that loss of DNA methylation in peri-ovulatory *Mll2^Gdf9^* cKO oocytes is not a widespread phenomenon. Merge and grayscale split channels (5-Me-C, FITC; DNA, propidium iodide, red) of single plane confocal sections are shown. Magnification: 800×.
(0.73 MB TIF)

Figure S4 **Generation and characterization of** ***Mll2*** **^tm2afst/tm2afst^ ((** ***Mll2*** **^tm2/tm2^) mice.** (A) Schematic representation of targeted mutation 2 *Mll2* ^tm2afst^ (denoted throughout as *Mll2* ^tm2^). An enhanced yellow fluorescence protein (eYFP) cassette was introduced in the N terminus end of *Mll2*, followed by a neomycin (neo) cassette flanked by two Frt (FLP recombination target) sites. FLPe (Flip)-mediated recombination resulted in a continuous reading frame from the authentic *Mll2* initiating codon, through eYFP and the residual FRT to the second amino acid of *Mll2*. (B) Mice carrying a single copy of *Mll2* ^tm2^ (*Mll2* ^tm2/+^) were intercrossed to obtain control (*Mll2* ^+/+^and *Mll2* ^tm2/+^) and experimental *Mll2* ^tm2/tm2^ mice. (C) Southern blot analysis of tail genomic DNA: a 3.8 kb band denotes the *Mll2* ^tm2/tm2^ allele, whereas a 3.0 kb band denotes the wild type (WT) allele. *Mll2* ^tm2/+^ mice are distinguished by the presence of the two bands. (D) Real time PCR analysis showing a small but significant decrease in *Mll2* mRNA levels in ovaries from *Mll2* ^tm2/tm2^ females (ovaries from three females were used in the analysis (*n* = 3); Student's *t* test, *p*<0.05). (E) Representative Western blot analysis showing a decrease in MLL2 levels in ovaries from *Mll2* ^tm2/tm2^ females; tubulin was used as loading control. (F) Fertility studies shown as cumulative number of pups over a 6-mo period. Females were mated to WT males. The cumulative number of pups produced by *Mll2* ^tm2/tm2^ females was lower than that of controls indicating subfertility (*n* = 10 per genotype). (G) PAS-stained ovaries from 3-wk-old mice. *Mll2* ^tm2/tm2^ ovaries show normal follicular development at this age. Original magnification: 50×. (H,I) Developmental potential of *Mll2* ^tm2/tm2^ embryos. Pre-pubertal (21-d-old) females were superovulated and mated to 6-wk-old *Mll2* ^tm2/tm2^ males, which showed no defects in fertility at this age (H) or WT males (I). Embryonic development was evaluated in vitro. Means ± S.E. from five independent experiments are shown (approximately 35 embryos per each female, from a total of 8–11 females per genotype, were used in the analysis). (H) When females were mated to *Mll2* ^tm2/tm2^ males, a significantly lower percentage of *Mll2* ^tm2/tm2^ embryos reached the blastocyst stage; embryos accumulated between 1C and 4C stages. In contrast, the majority of embryos from WT females developed to blastocysts. (I) When females were mated to WT males, few embryos from *Mll2* ^tm2/tm2^ females reached the blastocyst stage. Abbreviations: 1C, 1-cell; 2C, 2-cell; M, morula; Bl, blastocyst. ANOVA test; * *p*<0.05.
(1.40 MB TIF)

Figure S5 ***Mll2*** **^tm2/tm2^ embryos show defects in zygote genome activation (ZGA).** (A) Representative autoradiogram showing Transcription Required Complex (TRC) complex levels. Two-cell embryos underwent metabolic labeling to determine TRC synthesis, as a marker of ZGA. Note the reduction in TRC levels in *Mll2* ^tm2/tm2^ 2-cell embryos (Student's *t* test, * *p*<0.05) in *Mll2* ^tm2/tm2^ 2-cell embryo pools (three pools of embryos from six females per genotype were used in this experiment; *n* = 3); α-amanitin-(α-a)-treated embryos were used as negative controls. (B) Quantification of TRC complex levels. TRC was significantly reduced (Student's *t* test, * *p*<0.05) in *Mll2* ^tm2/tm2^ 2-cell embryo pools (three pools of embryos from six females per genotype were used in this experiment; *n* = 3).
(0.15 MB TIF)

Figure S6 ***Mll2*** **^tm2/tm2^ embryos show defects in H3K4 bulk methylation.** (A–C) Representative micrographs showing confocal microscopy analysis of H3K4me1(A), H3K4me2 (B), and H3K4me3 (C); a decrease in H3K4 di- and tri-methylation was observed in the female (f) pronucleus of *Mll2* ^tm2/tm2^ embryos; the male pronucleus (m) is negative for H3K4me2 and H3K4me3, as expected; polar bodies (PB) stain with all antibodies used, as previously reported. Merge and grayscale split channels (H3K3 methylation, FITC; DNA, propidium iodide, red) of single plane confocal sections are shown. (D–E) Western blot analysis of H3K4 methylation in chromatin fractions from zygotes. Three pools of 100 zygotes each from 3–4 females per genotype were used in three independent experiments. Total histones were used as internal loading control. (D) Representative micrographs of Western blots showing H3K4me1 and H3K4me3 levels. (E) Chemoluminescence quantification revealed a significant decrease in global H3K4me3 levels in *Mll2* ^tm2/tm2^ embryos; samples were normalized against total histones. Student's *t* test, * *p*<0.05.
(1.94 MB TIF)

Table S1 **List of primer sequences.**
(0.05 MB DOC)

Text S1 **Supplementary materials and methods.**
(0.08 MB DOC)

## Abbreviations

*Amhr2*: anti-Mullerian hormone receptor 2
*Bax*: Bcl2-associated X protein
*Casp6*: caspase 6
*Cdkn1a*: cyclin-dependent kinase inhibitor 1A
cKO: conditional knockout
EZH2: Enhancer of Zeste 2
*Gdf9*: growth differentiation factor 9
H3K4: histone 3 lysine 4
*Iap*: intracisternal A particle
MLL: mixed lineage leukemia
NSN: non-surrounding nucleolus
PMSG: pregnant mare serum gonadotropin
*Setd7*: SET domain lysine methyltransferase 7
SN: surrounded nucleolus
WT: wild type
YFP: yellow fluorescence protein
ZGA: zygotic genome activation
*Zp3*: zona pellucida 3
