# Supplementary figures and images for: MLL2 Is Required in Oocytes for Bulk Histone 3 Lysine 4 Trimethylation and Transcriptional Silencing
Source: PLoS Biol. 2010 Aug 17;8(8):e1000453. doi: 10.1371/journal.pbio.1000453 (PMC2923083; doi:10.1371/journal.pbio.1000453)

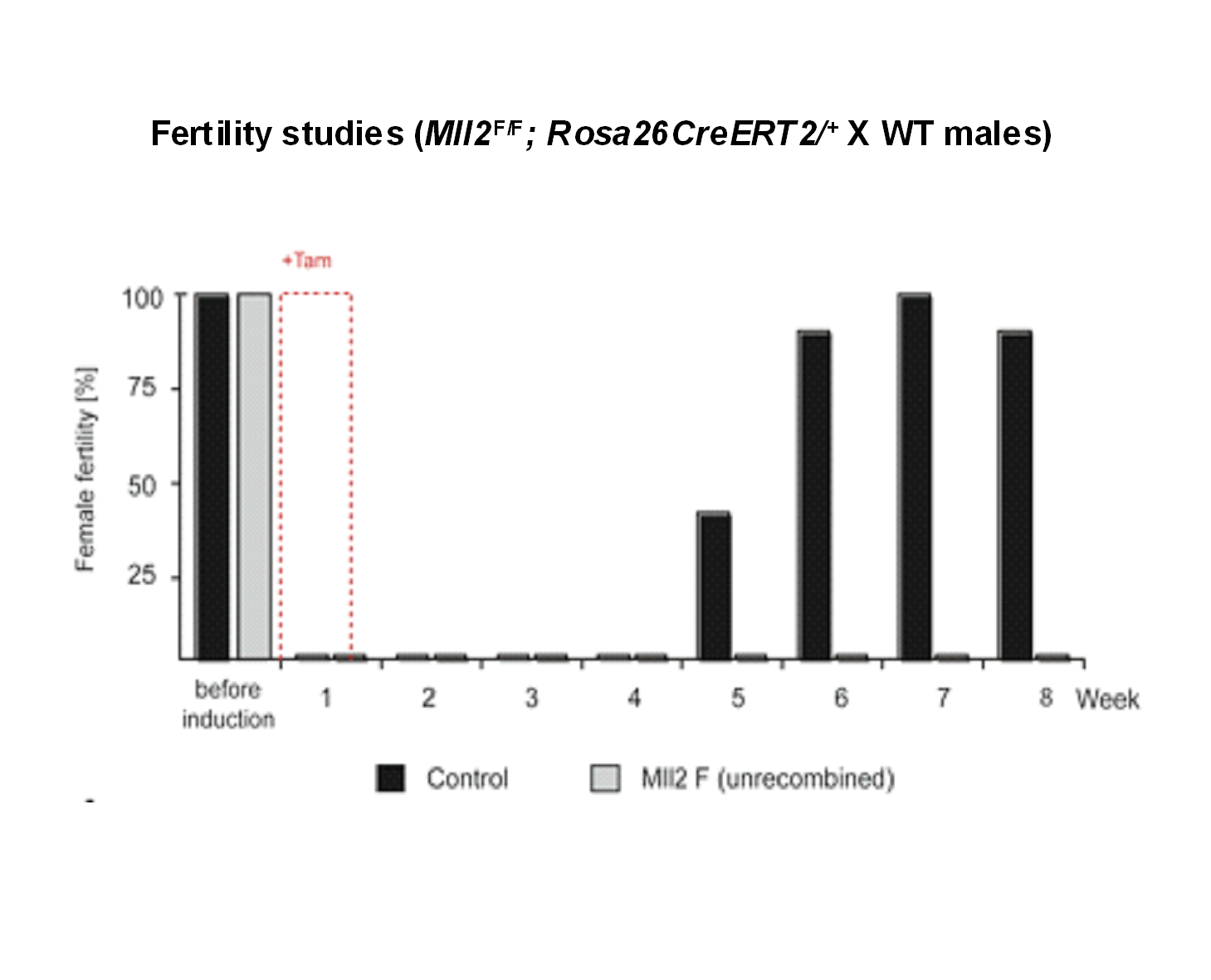

Supplement: Figure S1 — Loss of Mll2 in adult females using tamoxifen-induced Cre recombination leads to infertility. Mll2 F/F; Rosa26CreERT2/+ (Mll2F, conditional KO, grey bars) or Mll2 F/+; Rosa26CreERT2/+ (control, black bars) females (upper panel) were mated to WT males. Initially ten 2-mo-old adults of each genotype were mated to establish fertility (before induction), then they were treated with tamoxifen as described [38] and tested for fertility. The WT males (one male for each pair of tamoxifen treated females) were exchanged every week. Data show the number of successful litters. Note that tamoxifen treatment provokes infertility in females regardless of genotype. After treatment was discontinued, control animals fully recover fertility, whereas Mll2 conditional KO females remain infertile. (0.34 MB TIF) [file pbio.1000453.s001.tif]

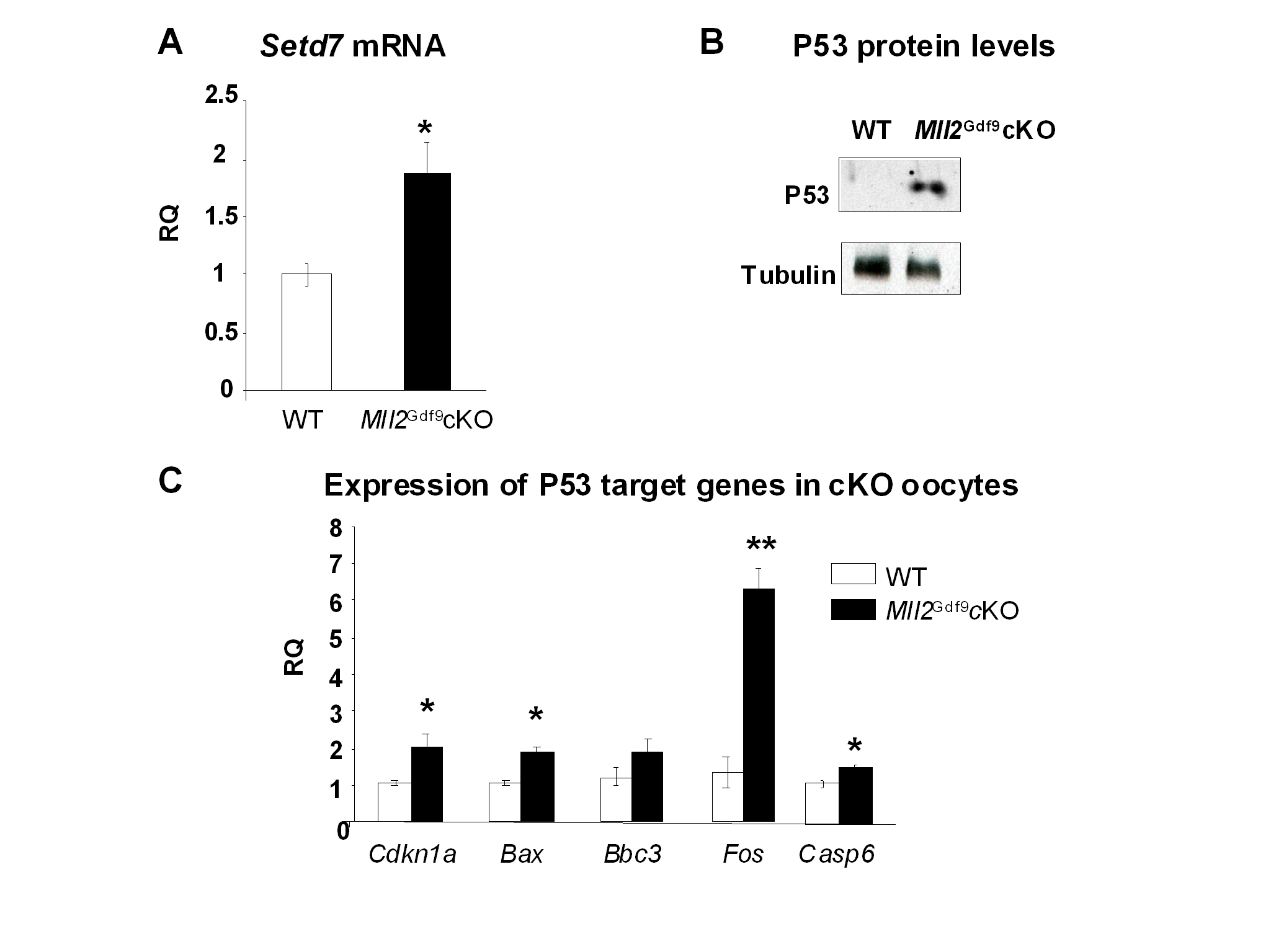

Supplement: Figure S2 — Mll2Gdf9 cKO oocytes show increased expression of the methyltransferase Setd7 , p53 stabilization, and expression of p53 downstream pro-apoptotic target genes. (A) QPCR analysis showed a significant increase in the methyltransferase Setd7 in Mll2Gdf9 cKO peri-ovulatory oocytes (Student's t test, * p<0.05; three pools of oocytes were used in the analysis (n = 3)). Gapdh was used as endogenous control. (B) Representative Western blot analysis showing increased p53 protein levels in Mll2Gdf9 cKO peri-ovulatory oocytes; tubulin was used as loading control. Note that p53 is normally absent in peri-ovulatory stage oocytes. (C) QPCR analysis showed a significant increase in the expression of p53 target genes,which are involved in apoptosis, including Bax, Cdkn1a, Fos, and Casp6 in Mll2Gdf9 cKO peri-ovulatory oocytes (Student's t test, * p<0.05; ** p<0.01; three pools of oocytes were used in the analysis (n = 3)). Gapdh was used as endogenous control. (0.18 MB TIF) [file pbio.1000453.s002.tif]

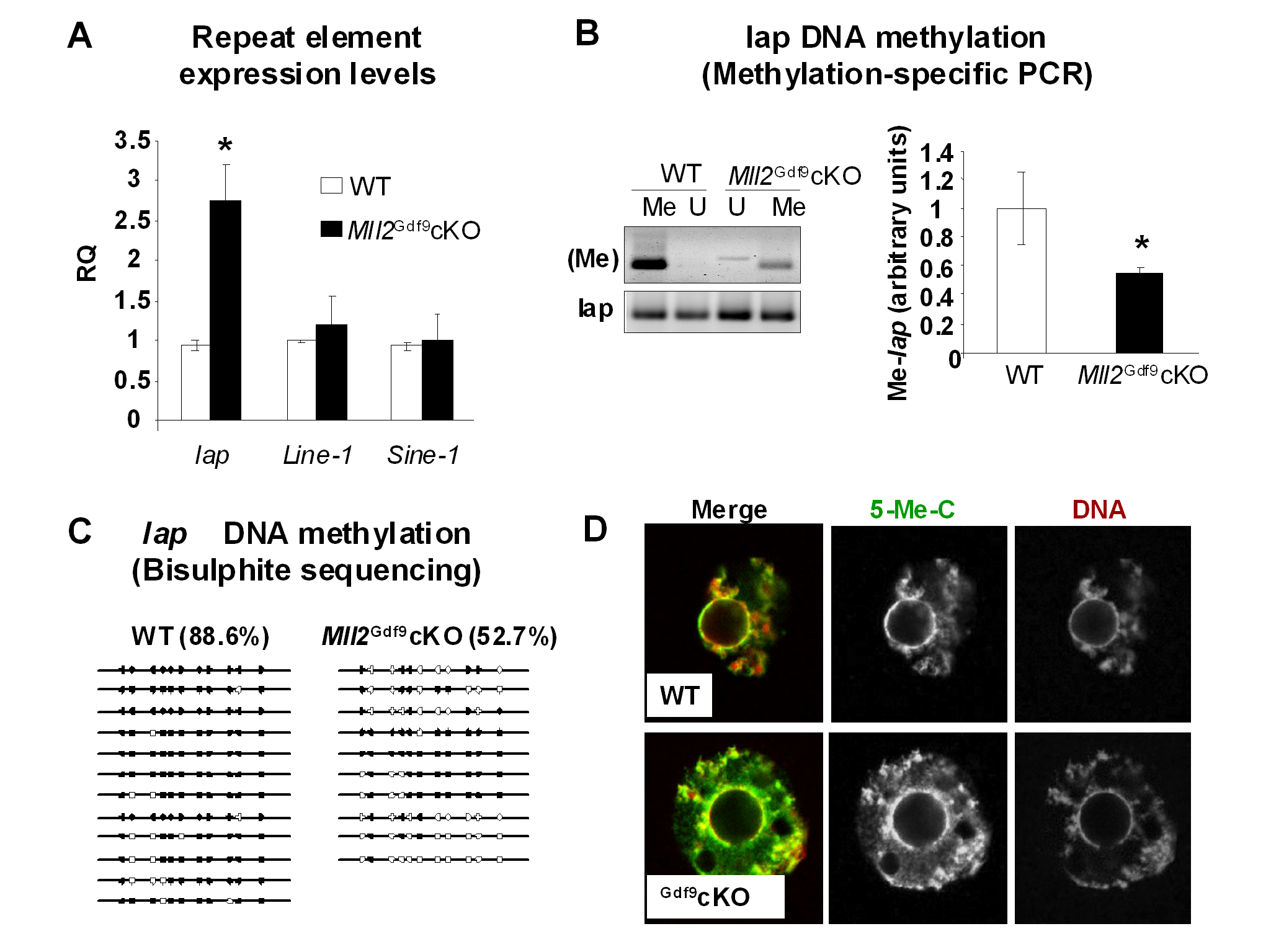

Supplement: Figure S3 — Mll2Gdf9 cKO oocytes display abnormal levels of the retrotransposable element Iap. (A) QPCR analysis of retrotransposon mRNA in isolated peri-ovulatory Mll2Gdf9cKO oocytes showed an increase (Student's t test, * p<0.05) in Iap (intracisternal A particle) but not in LINE-1 (Long Interspersed Nuclear Element 1, L1) or SINE-1 (Short Interspersed Nuclear Element); means ± S.E. are shown (three pools of 100 oocytes each were used in the analysis; n = 3). (B) Methylation-specific PCR analysis of the Iap promoter showed a significant decrease in CpG DNA methylation in peri-ovulatory Mll2Gdf9 cKO oocytes (Student's t test, * p<0.05); means ± S.E. are shown (three pools of 100 oocytes each were used in the analysis; n = 3). (C) Hypomethylation of Iap in peri-ovulatory Mll2Gdf9 cKO oocytes was confirmed by bisulphite sequencing. Methylated and unmethylated CpGs are shown as filled or open circles, respectively. (D) 5-methylcytosine (5-Me-C) staining and confocal microscopy analysis reveal that loss of DNA methylation in peri-ovulatory Mll2Gdf9 cKO oocytes is not a widespread phenomenon. Merge and grayscale split channels (5-Me-C, FITC; DNA, propidium iodide, red) of single plane confocal sections are shown. Magnification: 800×. (0.73 MB TIF) [file pbio.1000453.s003.tif]

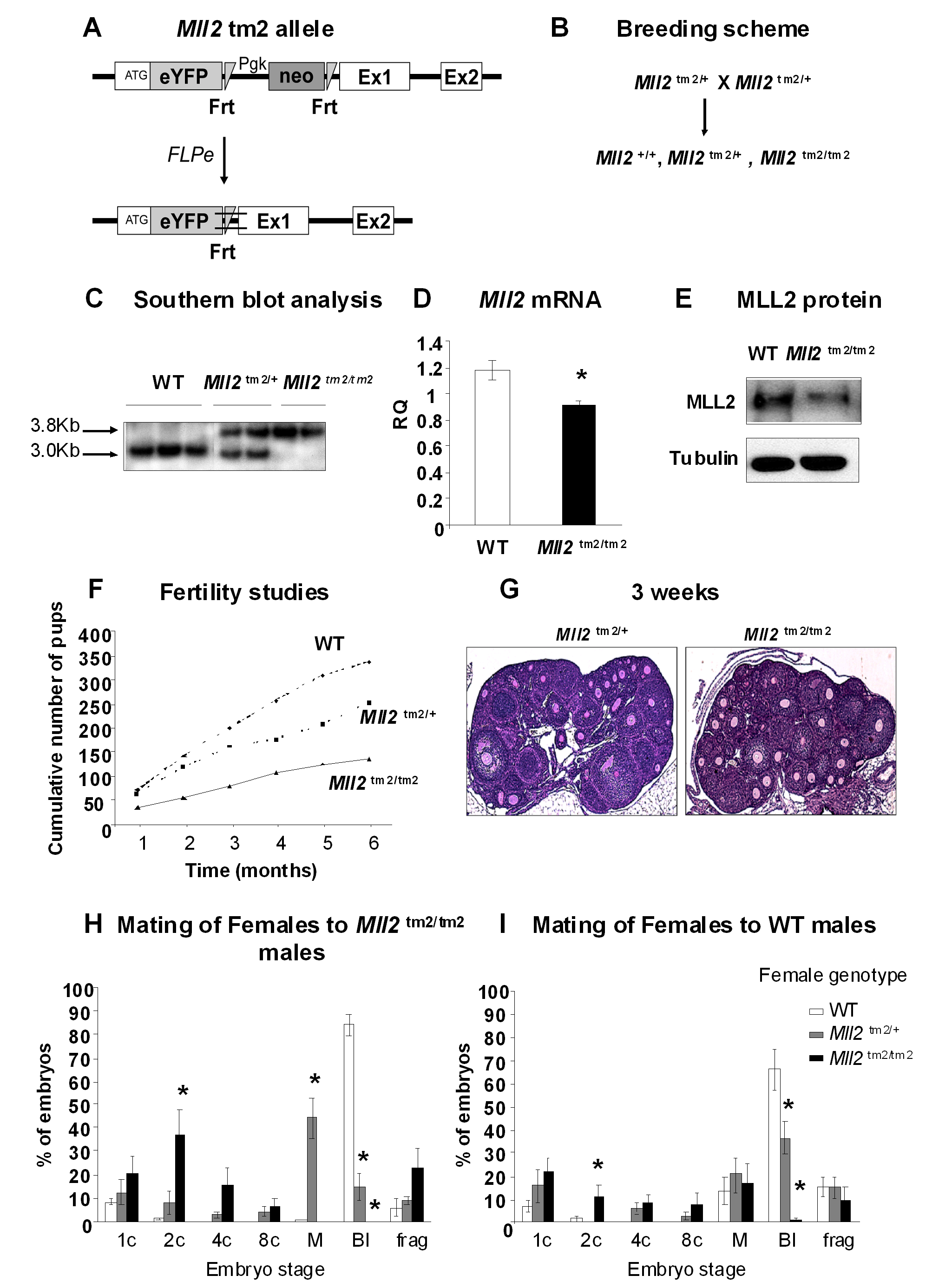

Supplement: Figure S4 — Generation and characterization of Mll2 tm2afst/tm2afst (( Mll2 tm2/tm2) mice. (A) Schematic representation of targeted mutation 2 Mll2 tm2afst (denoted throughout as Mll2 tm2). An enhanced yellow fluorescence protein (eYFP) cassette was introduced in the N terminus end of Mll2, followed by a neomycin (neo) cassette flanked by two Frt (FLP recombination target) sites. FLPe (Flip)-mediated recombination resulted in a continuous reading frame from the authentic Mll2 initiating codon, through eYFP and the residual FRT to the second amino acid of Mll2. (B) Mice carrying a single copy of Mll2 tm2 (Mll2 tm2/+) were intercrossed to obtain control (Mll2 +/+and Mll2 tm2/+) and experimental Mll2 tm2/tm2 mice. (C) Southern blot analysis of tail genomic DNA: a 3.8 kb band denotes the Mll2 tm2/tm2 allele, whereas a 3.0 kb band denotes the wild type (WT) allele. Mll2 tm2/+ mice are distinguished by the presence of the two bands. (D) Real time PCR analysis showing a small but significant decrease in Mll2 mRNA levels in ovaries from Mll2 tm2/tm2 females (ovaries from three females were used in the analysis (n = 3); Student's t test, p<0.05). (E) Representative Western blot analysis showing a decrease in MLL2 levels in ovaries from Mll2 tm2/tm2 females; tubulin was used as loading control. (F) Fertility studies shown as cumulative number of pups over a 6-mo period. Females were mated to WT males. The cumulative number of pups produced by Mll2 tm2/tm2 females was lower than that of controls indicating subfertility (n = 10 per genotype). (G) PAS-stained ovaries from 3-wk-old mice. Mll2 tm2/tm2 ovaries show normal follicular development at this age. Original magnification: 50×. (H,I) Developmental potential of Mll2 tm2/tm2 embryos. Pre-pubertal (21-d-old) females were superovulated and mated to 6-wk-old Mll2 tm2/tm2 males, which showed no defects in fertility at this age (H) or WT males (I). Embryonic development was evaluated in vitro. Means ± S.E. from five independent experiments ar [file pbio.1000453.s004.tif]

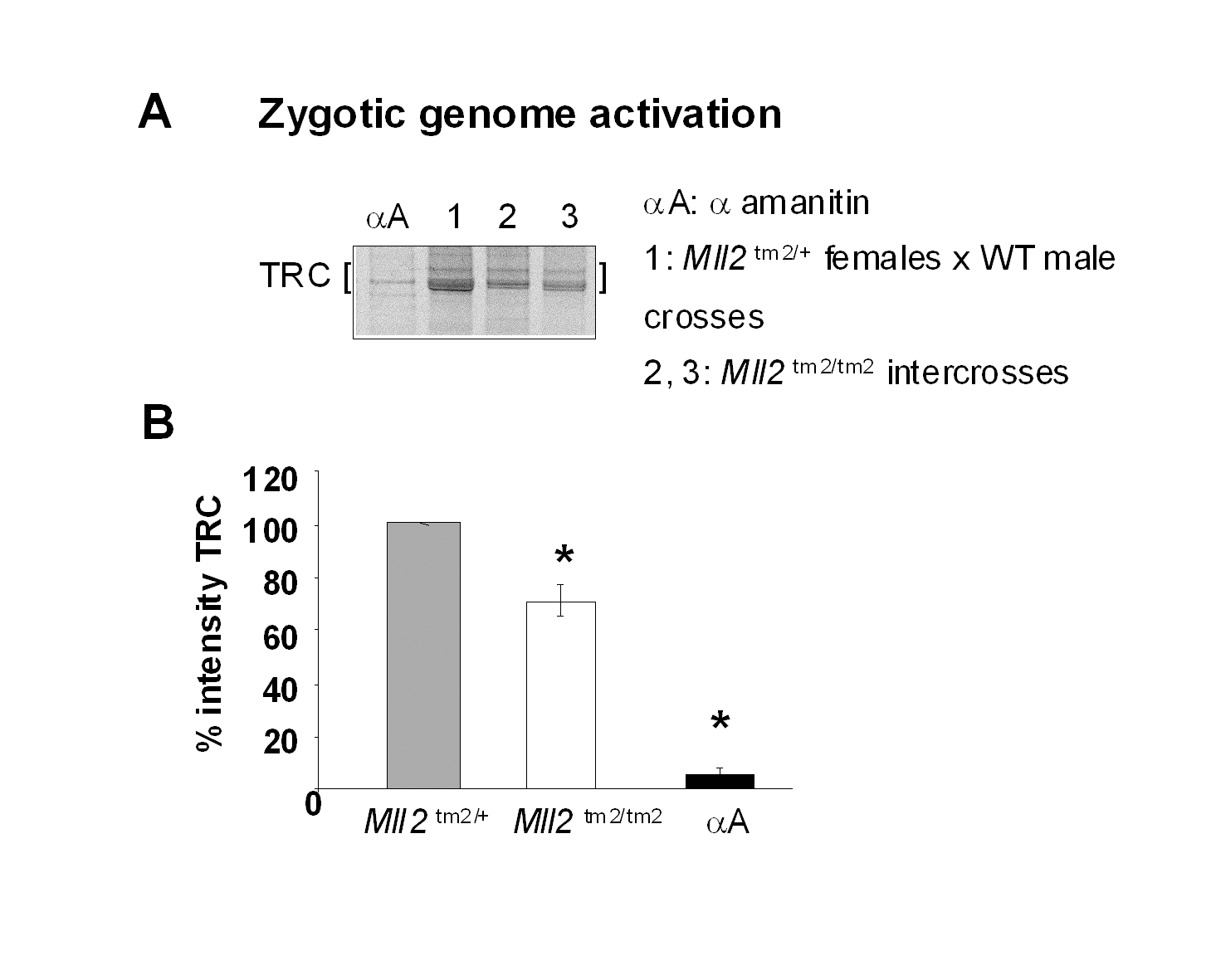

Supplement: Figure S5 — Mll2 tm2/tm2 embryos show defects in zygote genome activation (ZGA). (A) Representative autoradiogram showing Transcription Required Complex (TRC) complex levels. Two-cell embryos underwent metabolic labeling to determine TRC synthesis, as a marker of ZGA. Note the reduction in TRC levels in Mll2 tm2/tm2 2-cell embryos (Student's t test, * p<0.05) in Mll2 tm2/tm2 2-cell embryo pools (three pools of embryos from six females per genotype were used in this experiment; n = 3); α-amanitin-(α-a)-treated embryos were used as negative controls. (B) Quantification of TRC complex levels. TRC was significantly reduced (Student's t test, * p<0.05) in Mll2 tm2/tm2 2-cell embryo pools (three pools of embryos from six females per genotype were used in this experiment; n = 3). (0.15 MB TIF) [file pbio.1000453.s005.tif]

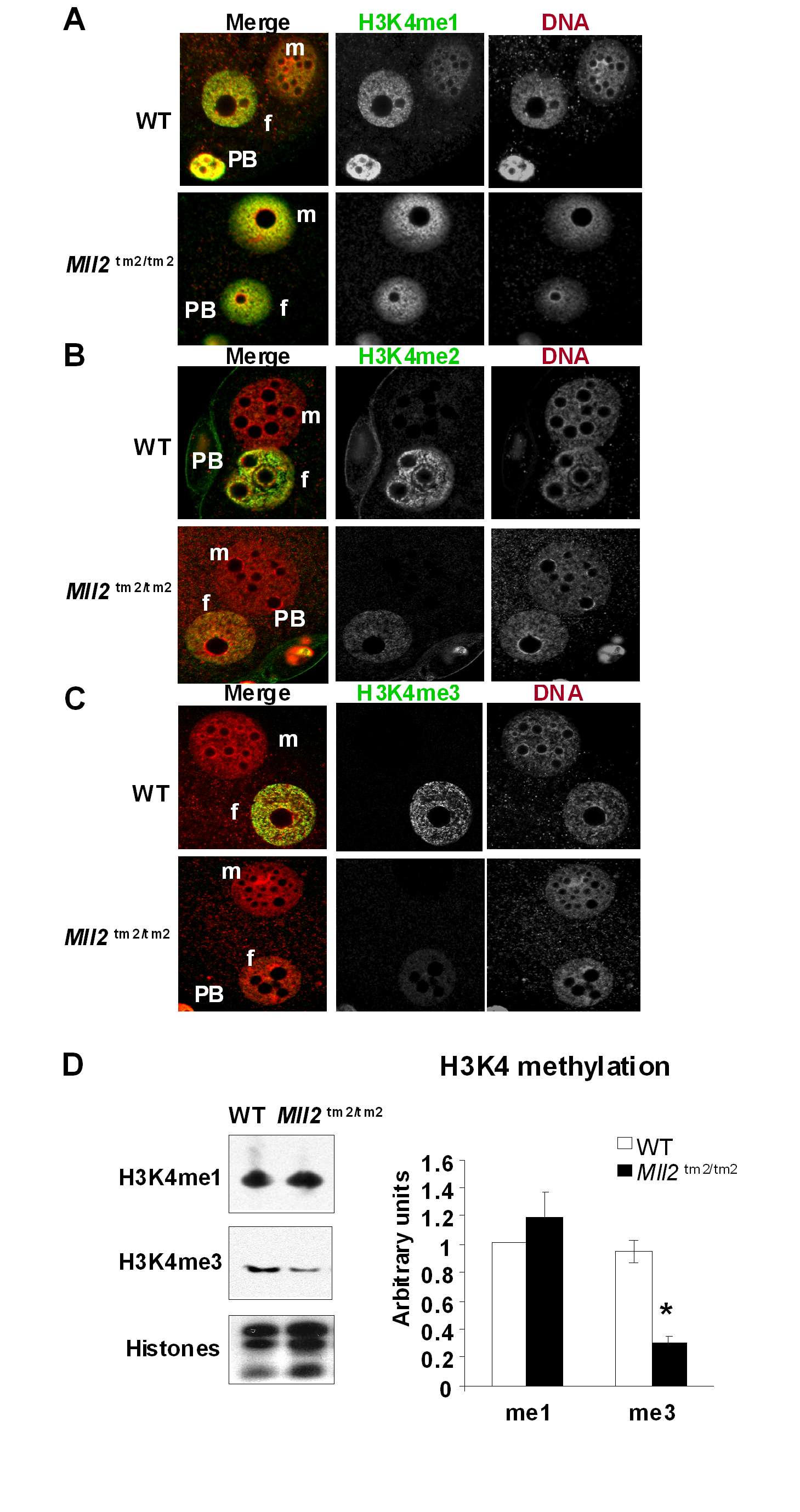

Supplement: Figure S6 — Mll2 tm2/tm2 embryos show defects in H3K4 bulk methylation. (A–C) Representative micrographs showing confocal microscopy analysis of H3K4me1(A), H3K4me2 (B), and H3K4me3 (C); a decrease in H3K4 di- and tri-methylation was observed in the female (f) pronucleus of Mll2 tm2/tm2 embryos; the male pronucleus (m) is negative for H3K4me2 and H3K4me3, as expected; polar bodies (PB) stain with all antibodies used, as previously reported. Merge and grayscale split channels (H3K3 methylation, FITC; DNA, propidium iodide, red) of single plane confocal sections are shown. (D–E) Western blot analysis of H3K4 methylation in chromatin fractions from zygotes. Three pools of 100 zygotes each from 3–4 females per genotype were used in three independent experiments. Total histones were used as internal loading control. (D) Representative micrographs of Western blots showing H3K4me1 and H3K4me3 levels. (E) Chemoluminescence quantification revealed a significant decrease in global H3K4me3 levels in Mll2 tm2/tm2 embryos; samples were normalized against total histones. Student's t test, * p<0.05. (1.94 MB TIF) [file pbio.1000453.s006.tif]
