# Supplementary material for: MLL2 Is Required in Oocytes for Bulk Histone 3 Lysine 4 Trimethylation and Transcriptional Silencing
Source: PLoS Biol. 2010 Aug 17;8(8):e1000453. doi: 10.1371/journal.pbio.1000453 (PMC2923083; doi:10.1371/journal.pbio.1000453)
Supplement: Table S1 — List of primer sequences. (0.05 MB DOC) [file pbio.1000453.s007.doc]

Table S1. Primers used in PCR and QPCR analyses

| **Genes** | **Forward Primers (5’-3’)** | **Reverse Primers (5’-3’)** |
| --- | --- | --- |
| **Mixed Lineage Leukemia 2 (*Mll2*)** | TGTTCGCATGAAAACGCCC | TGCAAGTGGCAGCAAAGGA |
| **B- cell leukemia/ lymphoma 2 (*Bcl2*)** | ATTGTGGCAGTCCCTTAGCCT | TGGCCGGCACACTTAACATT |
| **Phosphodiesterase 3a (*Pde3a*)** | CATCCACAAGCCCAGAGTGAA | TGGAAATGGCCTGCTTGTCT |
| **Cyclin B1 interacting protein 1 (*Ccnb1ip1*)** | AACCAGGTATGATCCCGCAGT | TCCACCACCTTGATTTCCAAC |
| **Bone morphogenetic protein 15 (*Bmp15*)** | AATGCCGGACCAAGCACTTA | TTGCGATTCCAGAGCTTCTGC |
| **Phosphatase and tensin homolog (*Pten*)** | ATAGCCCTAACCCCAAGAACG | TGAAACCTCCCATGTGCTGAT |
| **NLR family, pyrin domain containing 5 (*Nrlp5*)** | GTATGCCACGTTGGTGTTTCA | TGCCATCATGCACAAACCC |
| **Developmental pluripotency associated 3 (*Dpp3a*)** | TGTCGGTGCTGAAAGACCCTA | ATGGCTCACTGTCCCGTTCAA |
| **Steel factor receptor *(Ki*t)** | GGACCTGACGTTTGTCCCAAA | AATGCAGCCATGTACCGTCAC |
| **Y-box protein 2 (*Yx2*)** | TATCGAAGGCGGTTTGTGC | ATCACCCTCCAATGGTGCTGT |
| **SWI/SNF related, matrix associated, actin dependent regulator of chromatin, subfamily a, member 4 (*Smarca4*)** | CAACCACCCCTACATGTTCCA | TTCGTTGCACGGAGTTTGG |
| **SET domain containing 8 (*Setd8*)** | TCAGGAAGAGAACTCGGTTGC | GCTTCGTATAACGTTCCCGGT |
| **SET domain containing 1a (*Setd1a*)** | GCGACTCACATATGAACGGCT | TGCGCTTTGGAGTGCTTAGG |
| **Myeloid/lymphoid or mixed-lineage leukemia 3 (*Mll3*)** | CCCCAATTGCCCAACTATCT | TCGTAAGCGTTCCTTCCTCTCT |
| **SET domain containing 7 (*Setd7*)** | TCTCCAAGGCACCTATGTGGA | CCAACACACTCCGTGTCGATT |
| **WD repeat domain 5 (*Wdr5*)** | CCCACACCAGTTAAGCCAAAC | GCAGATGAACTTGCCAACCA |
| **Ash2 (absent, small, or homeotic)-like (Drosophila) (*Ash2l*)** | GCAAGCACTATTCGTCTGGCT | TTTGTAGGTGTCCGGCAGTGA |
| **Retinoblastoma binding protein 5 (*Rbbp5*)** | GGATGGAGAGCCAGAGCCTA | GTTGCCAATGCTCTTTTCCC |
| **K (lysine) acetyltransferase 5 (*Kat5/Tip60*)** | CACCCTCCAGGCAATGAAA | GGCCAGAAGACACAGGTTTTG |
| ***Iap* methylated (M)** | GGGAGTCGCGTTTATATTCGTC | ATTATTAAACGCGTTCTCACGCC |
| ***Iap* Unmethylated (U)** | GTTGGGAGTTGTGTTTATATTTGTT | TATTATTAAACACATTCTCACACCC |
