# Supplementary material for: MLL2 Is Required in Oocytes for Bulk Histone 3 Lysine 4 Trimethylation and Transcriptional Silencing
Source: PLoS Biol. 2010 Aug 17;8(8):e1000453. doi: 10.1371/journal.pbio.1000453 (PMC2923083; doi:10.1371/journal.pbio.1000453)
Supplement: Text S1 — Supplementary materials and methods. (0.08 MB DOC) [file pbio.1000453.s008.doc]

**Text S1**

**Generation of *Mll2* targeted mutation 2 (*Mll2* ^tm2afst^) mouse line and *Mll2* conditional KO mice**

We generated the targeted mutation 2 allele (*Mll2^tm2afst^*, denoted throughout *Mll2* ^tm2^) by introducing the yellow fluorescence protein (eYFP) cassette in frame with the first ATG of *Mll2* located in exon 1 (Figure S3A). The YFP reading frame was fused onto a Flip recognition target site (FRT)-flanked *PGK neo* polyadenylation cassette for gene targeting in E14 ES cells. After homologous recombination, the FRT cassette was removed by Flip (FLPe) recombination [1], resulting in a continuous reading frame from the authentic *Mll2* initiating codon, through YFP and the residual FRT to the second amino acid of *Mll2*. After FLP recombination, ES cells displayed a very weak YFP fluorescence that was entirely nucleoplasmic (data not shown). Mice were genotyped by Southern blot after double digestion with Asp701/HindIII, using a probe that recognizes *Mll2* exons 1, 2 and 3. The expected band sizes were 3.8kb and 3.0kb for mutant and wild type (WT) alleles, respectively. Conditional KO mice were generated by crossing growth differentiation factor 9 (*Gdf9*)*-Cre* [2], Zona pellucida 3 (*Zp3*)-*Cre* [3] or Anti-Mullerian Hormone receptor 2 (*Amhr2*)*-Cre* [4] mice and *Mll2* ^+/-^mice [5]. The progeny were crossed to *Mll2^F/F^* mice [5] to obtain experimental (*Mll2 ^FC/-^**Cre^+^* or cKO, for ‘conditional knock-out’) and control (*Mll2 ^F/-^Cre^-^* or *Mll2 ^F/-^*) littermates, where “F” refers to the allele after FLP recombination to restore wild type *Mll2* expression and “FC” refers to the F allele after Cre recombination to remove the floxed second exon, which provokes a frame shift mutation in exon 3 (Figures 1D, 1E, 7A and 9A). All mouse lines were maintained in the 129/C57BL/6 hybrid background. C57Bl/6J;129S6/SvEv hybrid female mice were also used as controls (WT). Genotyping from tail DNA was performed as described [2,4,5]. The decrease in *Mll2* levels in the various lines was confirmed by quantitative real time PCR (Fig.1F, Fig.7B, Fig.10B, Fig.S3D), and Western Blot analyses (Figures 1H, 9C, and S3E).

**Fertility studies, serum hormone levels, and histological analysis**

Eight to ten individually housed female mice were bred over a six-month period as described [6]. Serum FSH, estradiol, and LH were assayed by the University of Virginia Ligand Core Facility (Specialized Cooperative Centers Program in Reproductive Research NICHD/NIH U54 HD28934) [6]. Histological sections were stained with the periodic acid Schiff (PAS) reaction, and follicles were classified and counted as described [6]. Measurements were collected using the AxionVision 4.0 software (Carl Zeiss) and plotted as average number of follicles/mm^2^.

RT-PCR and quantitative real time PCR (QPCR) analyses

RNA from oocytes, embryos or granulosa cells was isolated using the PicoPure RNA kit (Arcturus, Molecular Devices) or the Rneasy Mini-Kit (Qiagen, Valencia, CA, USA). Sample preparation and reaction conditions were the same as reported [6]. Real-time QPCR was performed on the ABI Prism 7500 Sequence Detection System using primers described in Table S1. Primers against *Bax* (BCL2-associated X protein), *Trp53* (transformation related protein 53), *Fos* (FBJ osteosarcoma oncogene) and *Cdkn1a* (cyclin-dependent kinase inhibitor 1A (P21) were the same as reported [7]. Primer amplification efficiency and transcript levels were calculated as previously described [6]. The relative amount of target gene expression for each sample was plotted as the Mean ± SEM.

Cell collection and culture

To assess in vitro maturation, denuded oocytes were incubated in culture medium (MEM alpha medium; Invitrogen), 0.3% BSA (Sigma, St. Louis, MO, USA), 0.6 mM L-cysteine (Fisher Scientific, Pittsburgh, PA, USA, ascorbic acid (Sigma), and penicillin/streptomycin (Invitrogen) and scored after 16h in culture (MII). To evaluate *in vitro* development, females were mated and embryos were collected, cultured in M16 medium (Sigma), and scored at the indicated times. Data were collected from 12 females per genotype in 4 experiments.

Metabolic labeling and TRC complex detection

Metabolic labeling of 2-cell embryos and protein extraction were performed as previously reported [8] using embryos treated with 11 μg/ml of the RNA Pol II inhibitor alpha-amanitin (Sigma) as a negative control. Protein extracts were fractionated (20 embryo equivalents/lane), and ^35^S-radiolabelled proteins were detected as previously described [9]. The levels of TRC (an indicator of embryo genome activation) complex from at least 3 independent experiments were quantified by using Image J software (NIH).

#### Run-on assays

Transcriptional activity was determined in peri-ovulatory oocytes after 5-bromo uridine 5'-triphosphate (BrUTP; Sigma) incorporation [10], and 11μg/ml of α-amanitin was used to block RNA Pol II activity [11]. Detection of incorporated BrUTP was performed as described [10]. Chromatin configuration and transcriptional activity were simultaneously analyzed on a laser-scanning confocal microscope.

**Antibodies**

Primary antibodies: rabbit anti-H3K4me1/2/3, rabbit anti-acetyl H4K12, rabbit anti-pan-acetylH3, rabbit anti-H4K20me1, rabbit anti-H3K27me3, and rabbit H3K9me3 (Upstate Laboratories, Millipore); anti-β-tubulin (Sigma); rabbit anti-p53 (Santa Cruz Biotechnology), and rabbit anti-MLL2 [5]. Secondary antibodies: goat anti-mouse horseradish peroxidase (HRP) and goat anti-rabbit HRP (Jackson ImmunoResearch Laboratories Inc) and donkey anti-rabbit and goat anti-mouse Alexa 488 (Molecular Probes, Invitrogen).

**References**

1. Schaft J, Ashery-Padan R, van der Hoeven F, Gruss P, Stewart AF (2001) Efficient FLP recombination in mouse ES cells and oocytes. Genesis 31(1): 6-10.

2. Lan ZJ, Xu X, Cooney AJ (2004) Differential oocyte-specific expression of Cre recombinase activity in GDF-9-iCre, Zp3cre, and Msx2Cre transgenic mice. Biol Reprod 71(5): 1469-1474.

3. Lewandoski M, Wassarman KM, Martin GR (1997) Zp3-cre, a transgenic mouse line for the activation or inactivation of loxP-flanked target genes specifically in the female germ line. Curr Biol 7(2): 148-151.

4. Jamin SP, Arango NA, Mishina Y, Hanks MC, Behringer RR (2003) Genetic studies of the AMH/MIS signaling pathway for Mullerian duct regression. Mol Cell Endocrinol 211(1-2): 15-19.

5. Glaser S, Schaft J, Lubitz S, Vintersten K, van der Hoeven F et al. (2006) Multiple epigenetic maintenance factors implicated by the loss of Mll2 in mouse development. Development 133(8): 1423-1432.

6. Andreu-Vieyra C, Chen R, Matzuk MM (2008) Conditional deletion of the retinoblastoma (Rb) gene in ovarian granulosa cells leads to premature ovarian failure. Mol Endocrinol 22(9): 2141-2161.

7. Andreu-Vieyra C, Chen R, Matzuk MM (2007) Effects of granulosa cell-specific deletion of Rb in Inha-alpha null female mice. Endocrinology 148(8): 3837-3849.

8. Conover JC, Temeles GL, Zimmermann JW, Burke B, Schultz RM (1991) Stage-specific expression of a family of proteins that are major products of zygotic gene activation in the mouse embryo. Dev Biol 144(2): 392-404.

9. Poueymirou WT, Schultz RM (1987) Differential effects of activators of cAMP-dependent protein kinase and protein kinase C on cleavage of one-cell mouse embryos and protein synthesis and phosphorylation in one- and two-cell embryos. Dev Biol 121(2): 489-498.

10. De La Fuente R, Eppig JJ (2001) Transcriptional activity of the mouse oocyte genome: companion granulosa cells modulate transcription and chromatin remodeling. Dev Biol 229(1): 224-236.

11. Levey IL, Brinster RL (1978) Effects of alpha-amanitin on RNA synthesis by mouse embryos in culture. J Exp Zool 203(3): 351-360.
